# The asymptotic solutions for the motion of a charged symmetric gyrostat in the irrational frequency case

**DOI:** 10.1038/s41598-024-66866-5

**Published:** 2024-07-19

**Authors:** T. S. Amer, I. M. Abady, H. A. Abdo, H. F. El-Kafly

**Affiliations:** 1 Department of Mathematics, Faculty of Science, Tanta University, Tanta, 31527 Egypt; 2 Department of Mathematics and Computer Science, Faculty of Science, Suez University, Suez, 43518 Egypt; 3 Tanta Higher Institute of Engineering and Technology, Tanta, Egypt

**Keywords:** Rigid body motion, Nonlinear dynamics, Gyrostatic moment, Euler’s angels, Irrational frequency, Engineering, Mathematics and computing

## Abstract

The primary objective of this study is to explore the spatial rotary movements of a symmetrically charged rigid body (RB) that is rotating around a fixed point, akin to Lagrange’s scenario as a novel scenario where its center of mass experiences a slight displacement from the symmetry dynamic axis. The body’s movement is presumed to be affected by a gyrostatic moment and a force from an electromagnetic field, attributed to the presence of a located point charge on this axis. The regulating equations of motion that are pertaining to the equations Euler–Poisson are solved through the utilization of Poincaré’s small parameter method along with its adaptations when the scenario of irrational frequencies is considered. The three angles of Euler are derived and graphed to ascertain the body’s position at any point throughout the motion. The temporal evolutions of the achieved outcomes are drawn to showcase the significant impact of the selected parameters on the motion. The phase plane diagrams have been generated to illustrate the stability of the body during the motion. The novelty of studying the rotatory motion of a charged RB under these specific conditions lies in the intricate interplay of gyrostatic effects, magnetic interactions, and nonlinear dynamics. This research can push the boundaries of theoretical mechanics and provide valuable insights and tools for both theoretical advancements and practical applications. Moreover, the achieved results from this analysis can be utilized to improve the dynamic performance of diverse engineering applications, particularly those dependent on gyroscopic theory. This includes enhancing the functionality of satellites, compasses, submarines, and automatic pilots used in aircraft. Essentially, the findings have practical implications for optimizing the performance and stability of these systems.

## Introduction

One of the most significant problems that we deal with in our lives is the rotational motion of the RB. The significance of delving into this problem comes from its broad applicability across both the realms of physics and mathematics. Such problems are governed by both Euler and Poisson Equations ^1^ to determine the body’s angular velocity and its configuration at any time. A lot of research has studied this problem from different perspectives^2–17^. In^2^, an influenced motion of a RB by a combination of gyroscopic and potential forces with axisymmetric characteristics was investigated, in which two integrals of this problem were presented. In^3^, a new class of two-dimension integrable systems characterized by an additional third-degree integral in velocities was presented. The utility of this system extends to addressing the motion challenges of both a particle and a RB about a fixed point. Furthermore, four new integrable scenarios that capture the dynamics of a particle navigating in various settings, such as the plane, surfaces, and pseudosphere with variable curvature, was presented. In^4^, the authors provided a fourth integral pertaining to the motion of a RB around a stationary point, particularly when it is influenced by a GM. In^5^, the authors ascertain the comprehensive structure of the potential associated with the motion of a RB. This formulation enables the angular velocity to consistently reside within a principal plane of body inertia. The complexity of the problem’s solution is greatly reduced, making the process more straightforward and manageable.

In^6^, the emphasis is placed on examining the dynamics of a RB connected with an elastic spring, conceptualized as a pendulum model. The author explores numerical solutions through the application of Runge–Kutta algorithms. These solutions are graphed to clarify and examine the body’s behavior over time while taking into account a range of values for the physical parameters related to the body. In^7^, a novel methodology is introduced to address Poisson equations, particularly when the angular velocity components of RB rotation are dependent exclusively on functions of time. The underlying solution is expressed analytically and is dependent on two real-valued, time-dependent factors. In^8^, an innovative approach for the solution of Euler–Poisson’s system is proposed. This system, known for its analytical solution challenges, has seen successful exploration through this new solving procedure. In^9^, the Energy–Casimir approach is applied to investigate the stability of long-duration rotations in a heavy gyrostat. The authors established the necessary and sufficient conditions for certain types of permanent rotations. It is emphasized that the stability of these rotations depends on various parameters, including the geometry of the gyrostat and the GM. In^10^, it is focused on the dynamics of a charged RB in motion about a stationary point, equipped with a rotor affixed along a main axis. This study delves into the analysis of sufficient conditions leading to instability for this equilibrium, employing the method of linear approximation. Additionally, the author provides the stability’s necessary conditions through the application of the Energy–Casimir approach. The stability challenges associated with autonomous, non-conservative mechanical systems influenced by potential, gyroscopic, and dissipative forces are examined in^11^. The study delved into the impact of gyroscopic forces on the overall stability phenomenon, emphasizing the role they play. Furthermore, the study explored the potential for gyroscopic stabilization, often overlooked in the presence of complete dissipative forces but attainable through widespread damping.

In^12^, the small parameter approach was used to explore the solutions of the equations of motion (EOM) governing a rapidly spinning heavy solid about an ellipsoid of inertia main axis. It was demonstrated that, except for specific instances, the body tends to exhibit a pseudo-regular precession around the vertical axis in the initial approximation. Moreover, it was highlighted that, in this scenario, at least four out of the six initial conditions remain arbitrary. In^13^ and^14^, the investigation focuses on perturbed rotational movements of a RB, resembling regular precession at the satisfaction of Lagrange’s criteria, where the restoring torque is contingent upon the nutation angle. The analysis assumes a significant portion of the body’s angular velocity, closely aligned with the dynamic axis of symmetry, and perturbing torques that are comparatively minor in relation to the restoring torques. A specialized introduction of a small parameter, coupled with the application of the averaging approach, is employed. The resultant averaged EOM are obtained till the first and second approximations.

In^15^, the focus is on investigating the perturbed movement of a gyrostat revolving around a stationary point, characterized by a mass distribution closely resembling Lagrange’s configuration. The gyrostat is subject to the effects of multiple vectors, including a restoring torque, a GM, and a perturbing torque. The averaged EOM for the required approximations are derived. Furthermore, the angle of precession is computed according to the author’s desired approximation. In^16^, the authors investigated the rotational movement of a non-symmetric RB when subjected to constant torques in its body-fixed frame besides a first GM’s component. The analytic outcomes and their simulation are presented for two cases where a constant moment is considered along the middle and minor axes. These results are discussed in terms of separatrix surfaces, periodic or non-periodic solutions, equilibrium manifolds, and extreme values of periodic solutions. In^17,18^, the study concentrated on analyzing the movement of a dynamical model comprising a rotating gyro around a fixed point, similar to the criteria of Lagrange. The approach of averaging is employed to derive a more suitable averaging system for the EOM, considering a small parameter. As a result, the system’s analytical solutions are presented for two scenarios involving various forms of perturbing moments. The rotational behavior of a symmetric GM near Lagrange’s case as it experiences a vector of perturbed torque, a third projection of a GM, and a restoring torque vector has been examined in^18^. In^19^ and^20^, the Poincaré’s small parameter method is applied to present the analytical solutions for the motion of a RB in a Newtonian force field (NFF). In^21^, the technique of Krylov–Bogoliubov–Mitropolski is applied to obtain the solutions for a heavy RB about a fixed point. It is restricted that the body is influenced by a GM. In^22^, the movement of a symmetric RB around one of its principal axes is analyzed in a NFF alongside a GM where the second component is assumed to have zero value. The EOMs are examined with consideration of specific initial conditions. Through the utilization of the PSPM, solutions to these equations are obtained. Furthermore, periodic solutions are explored specifically for scenarios involving irrational frequencies. The rotational movement of a RB about a fixed point in a NFF, incorporating GM around the symmetry axis, is examined in^23^. The EOMs and their integrals are achieved, simplifying them into a two degrees-of-freedom quasi-linear autonomous system and one integral. The PSPM is utilized for the body in motion with a fixed point to explore the analytical periodic solutions. In^24^, both Taylor’s method and the average technique are applied to achieve the solutions of a RB, which contains a hollow filled with incompressible viscous liquid under the effects of GM, resistance torque, and body constant torques. In^25^, the EOMs of a RB with a fixed point were analyzed, which resembles a Lagrange’s gyroscope. A minor displacement of the center of gravity from the axis of symmetry was considered and used as a small parameter. In^26^, various cases were examined to determine the presence of periodic solutions for the RB motion. These solutions were expressed as either integer or fractional powers of the small parameter. The obtained outcomes in^27^ and^28^ generalized the work in^25^, where the case of the irrational frequency of a charge RB in the presence of the second and third components of a GM is examined. Whereas in^27^ the authors studied this problem when the action of the third component and the presence of NFF were considered. As for^28^, it considered the case where the third component of the GM only applied, but the body was charged due to an electromagnetic force field.

The study of dynamics within the realm of physics encompasses the intricate behaviors and interactions of particles and rigid bodies under various forces and conditions^29–32^. This compilation of works addresses significant aspects of these dynamics, with a particular focus on the influences of magnetic fields and torque. In^29^, the authors introduced a novel semi-analytical approach to solving the dynamics of charged particles subjected to magnetic fields with variable parameters. In^30^, the complex dynamics for the motion of rigid bodies around their center of mass have been explored. The provided analysis in this work covers a range of scenarios and conditions, making it an essential resource for understanding the behavior of rigid bodies in various fields. Under the influence of forces produced by the Barnett–London effect, the motion of a dynamically symmetric body around a fixed point in a uniform magnetic field was analyzed in^31^. The body’s steady rotations and regular precession were explained, and the problem has been simplified to a quadrature. The rotational evolution of heavy rigid bodies when subjected to varying restoring and perturbation torques was examined in^32^. This study provided insights into the nonlinear dynamics of rigid bodies, which expanded our understanding of how unsteady forces influence rotational motion.

The current work aims to investigate the spatial rotational movement of a symmetrically charged RB revolving about a fixed point analogous to Lagrange’s configuration when the body’s center of mass is slightly off the body’s axis of dynamic symmetry. It is considered that the body’s motion is influenced by both a force of the electromagnetic field and a GM. The fundamental EOM associated with Euler–Poisson’s equations are solved by applying PSPM along with its modifications in the case of irrational frequencies. The body’s Euler angles have also been calculated and graphically represented to determine the body’s position at any given moment during the motion. Plotting the time evolution of the obtained results demonstrates the substantial influence of the chosen gyrostat parameters on the movement. To demonstrate the body’s stability during motion, diagrams of phase plane are created and analyzed. The combination of nonlinear dynamics, magnetic interactions, and gyrostatic effects makes investigating the rotatory motion of a charged rigid body (RB) under these particular circumstances intriguing. The results of this research have the potential to expand the field of theoretical mechanics and offer useful tools and insights for both theoretical development and real-world applications. The applications of studying the rotatory motion of charged rigid bodies under the action of gyrostatic moments (GM) are vast and diverse, spanning from space technology and robotics to navigation, energy systems, and beyond. The insights gained from this research can lead to significant advancements in the design, control, and optimization of various systems that rely on precise rotational dynamics.

## Description of the problem

**Figure 1:**
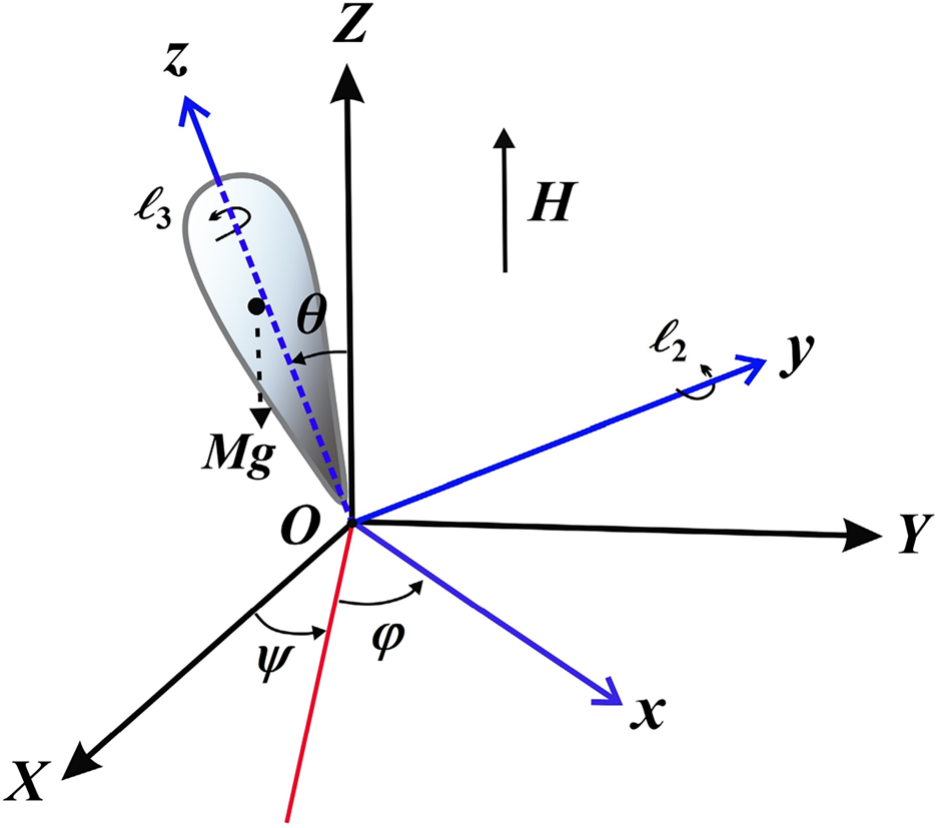
The problem’s physical model.

The rotary motion of a symmetrical RB of mass $M$ according to the criteria of Lagrange is investigated in this section. Through a fixed point $O$ inside the body, two frames have been taken into consideration. The first frame $OXYZ$ is the fixed in space, whereas the second one $Oxyz$ is taken to be fixed in the body and moving along its configuration (see Fig. 1). The body experiences a GM $\underline {\ell }$, with its components denoted as $\ell_{j} \,\,\,(j = 1,2,3)$, operating along the body’s inertia main axes $Ox,Oy,$ and $Oz$, in which $\ell_{1} = 0$. Furthermore, assuming that the body rotates under the action of a uniform electromagnetic field with the strength $H$, generated by a charge $e$ positioned on the axis $Oz$ at a distance $\ell^{*}$ from $O$ which makes an angle $\nu$ with the fixed axis $OZ$.

According to the aforementioned problem’s formulation, the controlling EOM of the RB can be stated as follows^1,27,28^

1 $$\begin{gathered} \underline{{\dot{h}}}_{O} + \underline{\omega } \times \underline{h}_{O} = \underline{L}_{O} , \hfill \\ \underline{{\dot{\hat{K}}}} + \underline{\omega } \times \underline{{\hat{K}}} = \underline{0} ; \hfill \\ \underline{h}_{O} = (Ap,Bq + \ell_{2} ,Cr + \ell_{3} ), \hfill \\ \underline{L}_{O} = \underline{r}_{c} \wedge Mg\underline{{\hat{K}}} + e(\underline{H} .\underline{{\ell^{*} }} )(\underline{\omega } \wedge \underline{{\ell^{*} }} ). \hfill \\ \end{gathered}$$

Here, $\underline{h}_{O}$ is the total angular momentum of the body and $\underline{L}_{O}$ is the total external moments affecting the body, $A,B,$ and $C$ are the body’s inertia moments along the main axes, respectively. According to the examined Lagrange’s case $A = B$. Within the definition of symbols in the preceding system (1), we find that $g$ is the gravitational acceleration, $\underline{r}_{c} = (x_{c} ,y_{c} ,z_{c} )$ refers to the position of the center of mass, $\underline{\omega } = (p,q,r)$ signifies the body’s angular velocity, $\underline{{\hat{K}}} = (\alpha ,\beta ,\gamma )$ denotes the unit vector along the downward direction of the fixed axis $OZ$, and dots over the parameters denote their derivatives regarding the time $t$.

Therefore, Eq. (1) can be represented as follows

2 $$\begin{gathered} A\dot{p} - (A - C)qr + (\ell_{3} - eH\ell^{*2} \cos \nu )q - \ell_{2} r = Mg(\gamma y_{c} - \beta z_{c} ), \hfill \\ A\dot{q} - (C - A)rp - (\ell_{3} - eH\ell^{*2} \cos \nu )p = Mg(\alpha z_{c} - \gamma x_{c} ), \hfill \\ C\dot{r} + \ell_{2} p = Mg(\beta x_{c} - \alpha y_{c} ), \hfill \\ \dot{\alpha } = r\beta - q\gamma , \hfill \\ \dot{\beta } = p\gamma - r\alpha , \hfill \\ \dot{\gamma } = q\alpha - p\beta . \hfill \\ \end{gathered}$$

The three first integrals respecting to system (2) are

3 $$\begin{gathered} A(p^{2} + q^{2} ) + Cr^{2} - 2Mg(x_{c} \alpha + y_{c} \beta + z_{c} \gamma ) = A(p_{0}^{2} + q_{0}^{2} ) + Cr_{0}^{2} \hfill \\ \quad \quad \;\quad \quad \, - 2Mg(x_{c} \alpha_{0} + y_{c} \beta_{0} + z_{c} \gamma_{0} ), \hfill \\ Ap\alpha + (Aq + \ell_{2} )\beta + [Cr + (\ell_{3} - eH\ell^{*2} \cos \nu )]\gamma = Ap_{0} \alpha_{0} + (Aq_{0} + \ell_{2} )\beta_{0} \hfill \\ \quad \quad + [Cr_{0} + (\ell_{3} - eH\ell^{*2} \cos \nu )]\gamma_{0} , \hfill \\ \alpha^{2} + \beta^{2} + \gamma^{2} = 1. \hfill \\ \end{gathered}$$

Here, $p_{0} ,q_{0} ,r_{0} ,\alpha_{0} ,\beta_{0} ,$ and $\gamma_{0}$ are, respectively, the values of $p,q,r,\alpha ,\beta ,$ and $\gamma$ at $t = 0$.

To apply the PSPM, let us define the following parameters

4 $$\begin{gathered} p = \sqrt \varepsilon \frac{{p_{1} }}{n},\quad q = \sqrt \varepsilon \frac{{q_{1} }}{n},\quad r = \frac{{r_{1} }}{n},\quad \alpha = \sqrt \varepsilon \alpha_{1} ,\quad \beta = \sqrt \varepsilon \beta_{1} ,\quad \hfill \\ \gamma = \gamma_{1} ,\quad \ell_{2} = \frac{A}{n}\sqrt \varepsilon \,\ell^{\prime}_{2} ,\quad \ell_{3} = \frac{A}{n}\ell^{\prime}_{3} ,\quad n = \sqrt {A/Mgz_{0} } ,\quad t = n\tau , \hfill \\ x_{c} = \sqrt \varepsilon z_{c} ,\quad y_{c} = 0,\quad a = (A - C)/A,\quad b = C/A,\quad \ell^{*} = \frac{n}{A}\ell^{\prime}. \hfill \\ \end{gathered}$$

Substituting (4) into systems (2) and (3) to get

5 $$\begin{aligned} \frac{{dp_{1} }}{d\tau } = & aq_{1} r_{1} + \left( {\ell^{\prime}_{3} - eH\ell^{{\prime}{2}} \cos \nu } \right)q_{1} + \ell^{\prime}_{2} r_{1} - \beta_{1} , \\ \frac{{dq_{1} }}{d\tau } = & - ap_{1} r_{1} + \left( {\ell^{\prime}_{3} - eH\ell^{{\prime}{2}} \cos \nu } \right)p_{1} + \alpha_{1} - \gamma_{1} , \\ \frac{{dr_{1} }}{d\tau } = & \frac{\varepsilon }{b}\left( {\beta_{1} - \ell^{\prime}_{2} p_{1} } \right), \\ \frac{{d\alpha_{1} }}{d\tau } = & r_{1} \beta_{1} - q_{1} \gamma_{1} , \\ \frac{{d\beta_{1} }}{d\tau } = & p_{1} \gamma_{1} - r_{1} \alpha_{1} , \\ \frac{{d\gamma_{1} }}{d\tau } = & \varepsilon \left( {q_{1} \alpha_{1} - p_{1} \beta_{1} } \right). \\ \end{aligned}$$

6 $$\begin{gathered} \varepsilon (p_{1}^{2} + q_{1}^{2} - 2\alpha_{1} ) + br_{1}^{2} - 2\gamma_{1} = \varepsilon \left( {p_{10}^{2} + q_{10}^{2} - 2\alpha_{10} } \right) + br_{10}^{2} - 2\gamma_{10} , \hfill \\ \varepsilon \left[ {p_{1} \alpha_{1} + (q_{1} + \ell^{\prime}_{2} )\beta_{1} } \right] + \left[ {br_{1} + (\ell^{\prime}_{3} - eH\ell^{{\prime}{2}} \cos \nu )} \right]\gamma_{1} = \varepsilon \left[ {p_{10} \alpha_{10} + (q_{10} + \ell^{\prime}_{2} )\beta_{10} } \right] \hfill \\ \quad \quad \quad \quad \quad \quad \quad \quad \quad \quad \quad \quad \quad \quad \quad \quad \quad \quad \quad \quad \;\; + \left[ {br_{10} + \left( {\ell^{\prime}_{3} - eH\ell^{{\prime}{2}} \cos \nu } \right)} \right]\gamma_{10} , \hfill \\ \varepsilon (\alpha_{1}^{2} + \beta_{1}^{2} ) + \gamma_{1}^{2} = 1. \hfill \\ \end{gathered}$$

## Procedure of the PSPM

In this section, we are going to investigate the procedure of the used method trying to obtain the problem’s solutions.

The first and third equations of system (6) allow us to write

7 $$r_{1} = r_{10} - \varepsilon f_{2} ,\quad \;\gamma_{1} = 1 - \varepsilon f_{1} ,$$

where

8 $$\begin{aligned} f_{1} = & \frac{1}{8}\left( {4F_{1} + \varepsilon F_{1}^{2} } \right) + \ldots , \\ f_{2} = & \frac{1}{{8br_{10} }}\left\{ {\left[ {4(F_{2} - F_{20} ) + \varepsilon (F_{1}^{2} - F_{10}^{2} )} \right] + \frac{\varepsilon }{{br_{10}^{2} }}(F_{2} - F_{20} )^{2} + \ldots } \right\}, \\ F_{1} = & \alpha_{1}^{2} + \beta_{1}^{2} , \\ F_{2} = & p_{1}^{2} + q_{1}^{2} - 2\alpha_{1} + F_{1} . \\ \end{aligned}$$

Here, $F_{d0} \,\,(d = 1,2)$ denotes the initial values of $F_{d0}$.

Substituting (7) into (5), one can reduce system (5) to the below first-order differential equation regarding the frequencies $\lambda_{d}$

9 $$\begin{aligned} \frac{{dp_{2} }}{dt} = & \Omega \ell^{\prime}_{2} r_{10} + \lambda_{1} q_{2} + \varepsilon G_{1} , \\ \frac{{dq_{2} }}{dt} = & - \lambda_{1} p_{2} + \varepsilon G_{2} , \\ \frac{{d\alpha_{2} }}{dt} = & - \xi \ell^{\prime}_{2} r_{10} + \lambda_{2} \beta_{2} + \varepsilon G_{3} , \\ \frac{{d\beta_{2} }}{dt} = & - \lambda_{2} \alpha_{2} + \varepsilon G_{4} , \\ \end{aligned}$$

where

10 $$\begin{aligned} p_{1} = & p_{2} + h\alpha_{2} + c_{1} , \\ q_{1} = & q_{2} + h\beta_{2} , \\ \alpha_{1} = & \Omega \alpha_{2} + \xi p_{2} + c_{2} , \\ \beta_{1} = & \Omega \beta_{2} + \xi q_{2} , \\ \omega = & \frac{1}{2}\left[ { - (br_{10} + \ell_{3}^{\prime } - eH\ell^{\prime 2} \cos \nu ) \pm \sqrt {(br_{10} + \ell_{3}^{\prime } - eH\ell^{\prime 2} \cos \nu )^{2} + 4)} } \right], \\ h = & 1/ \pm \sqrt {(br_{10} + \ell_{3}^{\prime } - eH\ell^{\prime 2} \cos \nu )^{2} + 4)} , \\ c_{1} = & r_{10} /(1 - ar_{10}^{2} + \ell^{\prime}_{3} r_{10} - r_{10} eH\ell^{\prime 2} \cos \nu ), \\ c_{2} = & c_{1} /r_{10} , \\ \lambda_{1} = & h - \xi hr_{10} + \Omega (ar_{10} - \ell^{\prime}_{3} + \xi + eH\ell^{\prime 2} \cos \nu ), \\ \lambda_{2} = & h( - a\xi r_{10} + \xi \ell^{\prime}_{3} - eH\ell^{\prime 2} \cos \nu - 1) + \Omega (\xi + r_{10} ), \\ G_{1} = & - \Omega f_{2} (aq_{1} + \ell^{\prime}_{2} ) + h(f_{2} \beta_{1} - q_{1} f_{1} ), \\ G_{2} = & \Omega (ap_{1} f_{2} + f_{1} ) + h(p_{1} f_{1} - \alpha_{1} f_{2} ), \\ G_{3} = & \xi (aq_{1} + \ell^{\prime}_{2} )f_{2} - (\beta_{1} f_{2} - q_{1} f_{1} ), \\ G_{4} = & - \xi (f_{1} + ap_{1} f_{2} ) - (p_{1} f_{1} - \alpha_{1} f_{2} ), \\ \Omega = & (1 + \xi h). \\ \end{aligned}$$

For $\lambda_{d} (d = 1,2)$, if we consider that $\lambda_{1} /\lambda_{2}$ is a rational number by a suitable selection of $r_{0}$, then the solution of the generating system of Eq. (9) is periodic with period $T_{0} = 2\pi n_{d} /\lambda_{d}$.

However, we proceed by reformulating the problem to estimate the solution of system (9) with period $\tau_{0} (\varepsilon )$ according to an extremely small value of $\varepsilon$. At $\varepsilon = 0$, the periodic solution of the generated system of (8) exhibits a period $T_{0}$. So, keep in mind the following substitution

11 $$\tau = (1 + \varepsilon \rho )T,$$

where $\rho$ depends on $\varepsilon$, which can be determined subsequently.

Applying the aforementioned transformation of (11) to system (9), we have

12 $$\begin{aligned} \frac{{dp_{2} }}{dT} = & \Omega \ell^{\prime}_{2} r_{10} + \lambda_{1} q_{2} + \varepsilon H_{1} , \\ \frac{{dq_{2} }}{dT} = & - \lambda_{1} p_{2} + \varepsilon H_{2} , \\ \frac{{d\alpha_{2} }}{dT} = & - \xi \ell^{\prime}_{2} r_{10} + \lambda_{2} \beta_{2} + \varepsilon H_{3} , \\ \frac{{d\beta_{2} }}{dT} = & - \lambda_{2} \alpha_{2} + \varepsilon H_{4} , \\ \end{aligned}$$

where

13 $$\begin{aligned} H_{1} = & [\Omega \ell^{\prime}_{2} r_{10} + \lambda_{1} q_{2} ]\rho + (1 + \varepsilon \rho )G_{1} , \\ H_{2} = & - \lambda_{1} p_{2} \rho + (1 + \varepsilon \rho )G_{2} , \\ H_{3} = & [ - \xi \ell^{\prime}_{2} r_{10} + \lambda_{2} \beta_{2} ]\rho + (1 + \varepsilon \rho )G_{3} , \\ H_{4} = & - \lambda_{2} \alpha_{2} \rho + (1 + \varepsilon \rho )G_{4} , \\ H_{j} = & H_{j}^{(1)} + \varepsilon H_{j}^{(2)} + \varepsilon^{2} H_{j}^{(3)} + ....,\,\,(j = 1,2,3,4) \\ \end{aligned}$$

To achieve the desired solutions, it is imperative that system (12) receives considerable attention. Hence, by further differentiating of system (12) again, we can derive the below forms of nonlinear differential equations from second-order

14 $$\begin{aligned} \frac{{d^{2} p_{2} }}{{dT^{2} }} + \lambda_{1}^{2} p_{2} = & \varepsilon (\dot{H}_{1} + \lambda_{1} H_{2} ), \\ \frac{{d^{2} q_{2} }}{{dT^{2} }} + \lambda_{1}^{2} q_{2} = & \varepsilon (\dot{H}_{2} - \lambda_{1} H_{1} ) - \lambda_{1} \Omega \ell^{\prime}_{2} r_{10} , \\ \frac{{d^{2} \alpha_{2} }}{{dT^{2} }} + \lambda_{2}^{2} \alpha_{2} = & \varepsilon (\dot{H}_{3} + \lambda_{2} H_{4} ), \\ \frac{{d^{2} \beta_{2} }}{{dT^{2} }} + \lambda_{2}^{2} \beta_{2} = & \varepsilon (\dot{H}_{4} - \lambda_{2} H_{3} ) + \lambda_{2} \xi \ell^{\prime}_{2} r_{10} , \\ \end{aligned}$$

Consequently, we may assume the solutions of the aforementioned system as follows

15 $$\begin{aligned} p_{2} (T,\varepsilon ) = & M_{1} \cos \lambda_{1} T + M_{2} \sin \lambda_{1} T + \mathop {\sum , }\limits_{1} \\ q_{2} (T,\varepsilon ) = & M_{2} \cos \lambda_{1} T - M_{1} \sin \lambda_{1} T - \frac{{\Omega \ell^{\prime}_{2} r_{10} }}{{\lambda_{1} }} + \mathop {\sum , }\limits_{2} \\ \alpha_{2} (T,\varepsilon ) = & M_{3} \cos \lambda_{2} T + \mathop {\sum , }\limits_{3} \\ \beta_{2} (T,\varepsilon ) = & \frac{{\xi \ell^{\prime}_{2} r_{10} }}{{\lambda_{2} }} - M_{3} \sin \lambda_{2} T + \mathop {\sum , }\limits_{4} \\ \end{aligned}$$

where

$$\mathop {\sum = }\limits_{j} \mathop {\sum {\varepsilon^{n} } }\limits_{n = 1}^{\infty } C_{j}^{(n)} (T);\,\,\,\,\,\,(j = 1,2,3,4).$$

Furthermore, considering the subsequent initial criteria

16 $$\begin{aligned} p_{2} (0,\varepsilon ) = & M_{1} = M_{1}^{(0)} + m_{1} , \\ q_{2} (0,\varepsilon ) = & M_{2} - \frac{{\Omega \ell^{\prime}_{2} r_{10} }}{{\lambda_{1} }} = M_{2}^{(0)} - \frac{{\Omega \ell^{\prime}_{2} r_{10} }}{{\lambda_{1} }} + m_{2} , \\ \alpha_{2} (0,\varepsilon ) = & M_{3} = M_{3}^{(0)} + m_{3} , \\ \beta_{2} (0,\varepsilon ) = & \frac{{\xi \ell^{\prime}_{2} r_{10} }}{{\lambda_{2} }}, \\ \end{aligned}$$

where $m_{j} (j = 1,2,3,4)$ and $M_{i}^{(0)} (i = 1,2,3)$ represent the perturbed and unperturbed terms of $M_{i}$, respectively.

Observably, the solutions of system (9) exhibiting a period $T_{0}$ match the periodic solutions of the equations in (14) with a period $T_{1} = (1 + \varepsilon \rho )T_{0}$. Assuming $\rho = (\rho_{0} + m_{4} )$ to derive the solutions (15) in their periodic forms, and considering $m_{j} = m_{j} (\varepsilon )$ where $m_{j}$ at $\varepsilon = 0$.

Following the procedure outlined in the SPMP, the initial criteria can be adjusted to align with arbitrary constants for the system’s generating solutions. Making use of (10) and (8) to obtain

17 $$\begin{aligned} F_{1}^{(0)} = & c_{2}^{2} + \Omega^{2} M_{3}^{2} + \xi^{2} (M_{1}^{2} + M_{2}^{2} ) + 2\xi \Omega M_{3} \left[ {M_{1} \cos (\lambda_{1} - \lambda_{2} )T + M_{2} \sin (\lambda_{1} - \lambda_{2} )T} \right] \\ & + 2c_{2} \Omega M_{3} \cos \lambda_{2} T + 2\xi c_{2} \left[ {M_{1} \cos \lambda_{1} T + M_{2} \sin \lambda_{1} T} \right] \\ & + 2\xi \Omega^{2} \ell^{\prime}_{2} r_{10} \left( {\frac{1}{{\lambda_{1} }} + \frac{1}{{\lambda_{2} }}} \right)M_{3} \sin \lambda_{2} T + (\xi \ell^{\prime}_{2} r_{10} )^{2} \Omega^{2} \\ & \times \left( {\frac{1}{{\lambda_{1} }} - \frac{1}{{\lambda_{2} }}} \right)^{2} + 2\xi^{2} \ell^{\prime}_{2} r_{10} \Omega \left( {\frac{1}{{\lambda_{1} }} - \frac{1}{{\lambda_{2} }}} \right)\left( {M_{1} \sin \lambda_{1} T - M_{2} \cos \lambda_{1} T} \right). \\ \end{aligned}$$

$$\begin{aligned} F_{2}^{(0)} = & c_{1}^{2} + (M_{1}^{2} + M_{2}^{2} ) + 2(c_{1} - \xi )[M_{1} \cos \lambda_{1} T + M_{2} \sin \lambda_{1} T] + h^{2} M_{3}^{2} \\ & + 2hM_{3} [M_{1} \cos (\lambda_{1} - \lambda_{2} )T + M_{2} \sin (\lambda_{1} - \lambda_{2} )T] - 2c_{2} + 2(c_{1} h - \xi - 1) \\ & \times M_{3} \cos \lambda_{2} T + \left\{ {2\ell^{\prime}_{2} r_{10} \left[ {\frac{\Omega }{{\lambda_{1} }} - \frac{\xi h}{{\lambda_{2} }}} \right]} \right\}\left[ {(M_{1} \sin \lambda_{1} T - M_{2} \cos \lambda_{1} T) + hM_{3} \sin \lambda_{2} T} \right] \\ & + (\ell^{\prime}_{2} r_{10} )^{2} \left[ {\frac{\Omega }{{\lambda_{1} }} - \frac{\xi h}{{\lambda_{2} }}} \right]^{2} + F_{1}^{(0)} . \\ \end{aligned}$$

Functions $G_{j} (j = 1,2,3,4)$ in system (10) may have the forms

18 $$\begin{aligned} G_{1} = & L_{1} q_{2} + L_{2} \beta_{2} + L_{3} , \\ G_{2} = & - L_{1} p_{2} - L_{2} \alpha_{2} + L_{4} , \\ G_{3} = & L_{5} q_{2} + L_{6} \beta_{2} + L_{7} , \\ G_{4} = & - L_{5} p_{2} - L_{6} \alpha_{2} + L_{8} , \\ \end{aligned}$$

where $L_{s} (s = 1,2,3,...,8)$ can be given in terms of $f_{d} \,\,\,(d = 1,2)$ as in (Appendix 1).

Therefore, one can write the non-perturbation terms of $L_{s}$ in the following way

19 $$\begin{aligned} L_{s}^{(0)} = & \left[ {S_{k1} + S_{k2} (M_{1}^{2} + M_{2}^{2} ) + S_{k3} M_{3}^{2} } \right] + S_{k4} \left( {M_{1} \cos \lambda_{1} T + M_{2} \sin \lambda_{1} T} \right) \\ & + S_{k5} M_{3} \cos \lambda_{2} T + S_{k6} M_{3} \left[ {M_{1} \cos (\lambda_{1} - \lambda_{2} )T + M_{2} \sin (\lambda_{1} - \lambda_{2} )T} \right] \\ & + S_{k7} \left( {M_{1} \sin \lambda_{1} T - M_{2} \cos \lambda_{1} T} \right) + S_{k8} M_{3} \sin \lambda_{2} T, \\ \end{aligned}$$

where $S_{k\eta }$ are the elements of the square matrix $\left\| {S_{k\eta } } \right\|(k,\eta = 1,2,3,...,8)$. To obtain these elements, one can substitute $F_{d}^{(0)}$ into (8) to calculate $f_{d}^{(0)}$. So, $S_{1\eta } ,S_{2\eta } ,...,S_{8\eta }$ can be easily calculated (see Appendix 2).

Referring to the solutions (15), the complete determination of these solutions is achievable, provided when the parameters $C_{j}^{(n)} (T)(j = 1,2,3,4)$ are obtained. As a result, we can simply create the system that specifies these parameters in (15) according to the substitution of (15) into (12) and then equating the like coefficients of $\varepsilon$ in each side to get

20 $$\begin{aligned} \frac{{dC_{1}^{(n)} }}{dT} = & \lambda_{1} C_{2}^{(n)} (T) + H_{1}^{(n)} (T), \\ \frac{{dC_{2}^{(n)} }}{dT} = & - \lambda_{1} C_{1}^{(n)} (T) + H_{2}^{(n)} (T), \\ \frac{{dC_{3}^{(n)} }}{dT} = & \lambda_{2} C_{4}^{(n)} (T) + H_{3}^{(n)} (T), \\ \frac{{dC_{4}^{(n)} }}{dT} = & - \lambda_{2} C_{3}^{(n)} (T) + H_{4}^{(n)} (T). \\ \end{aligned}$$

In addition to the following initial circumstances

21 $$C_{j}^{(n)} (0) = 0\quad (j = 1,2,3,4),$$

where $H_{j}^{(n)} (T)$ are functions that can be found once $C_{j}^{(\chi )}$ have been estimated at $\chi < n$.

When $$n = 1:$$

Upon substituting the expression of $H_{j}$ from Eq. (12) into Eq. (20), one can then proceed to compare the coefficients of the distinct powers of $\varepsilon$ on either side. Differentiating the resulting equations with respect to $T$ will allow us to obtain equations that determine $C_{j}^{(1)} (T)$ in the form

22 $$\begin{aligned} \frac{{d^{2} C_{1}^{(1)} }}{{dT^{2} }} + \lambda_{1}^{2} C_{1}^{(1)} (T) = & Q_{11} (T), \\ \frac{{d^{2} C_{2}^{(1)} }}{{dT^{2} }} + \lambda_{1}^{2} C_{2}^{(1)} (T) = & Q_{21} (T), \\ \frac{{d^{2} C_{3}^{(1)} }}{{dT^{2} }} + \lambda_{2}^{2} C_{3}^{(1)} (T) = & Q_{31} (T), \\ \frac{{d^{2} C_{4}^{(1)} }}{{dT^{2} }} + \lambda_{2}^{2} C_{4}^{(1)} (T) = & Q_{41} (T), \\ \end{aligned}$$

where

$$\begin{aligned} Q_{11} (T) = & - 2\lambda_{1} (\lambda_{1} \rho_{0} + L_{1}^{(0)} )p_{2}^{(0)} - (\lambda_{1} + \lambda_{2} )L_{2}^{(0)} \alpha_{2}^{(0)} + \frac{{dL_{1}^{(0)} }}{dT}q_{2}^{(0)} + \frac{{dL_{2}^{(0)} }}{dT}\beta_{2}^{(0)} + \frac{{dL_{3}^{(0)} }}{dT} + \lambda_{1} L_{4}^{(0)} , \\ Q_{21} (T) = & - 2\lambda_{1} [\Omega \ell^{\prime}_{2} r_{10} \rho_{0} + (\lambda_{1} \rho_{0} + L_{1}^{(0)} )q_{2}^{(0)} ] - \ell^{\prime}_{2} r_{10} [\Omega L_{1}^{(0)} - \xi L_{2}^{(0)} ] \\ & - (\lambda_{1} + \lambda_{2} )L_{2}^{(0)} \beta_{2}^{(0)} - \lambda_{1} L_{3}^{(0)} - \frac{{dL_{1}^{(0)} }}{dT}p_{2}^{(0)} - \frac{{dL_{2}^{(0)} }}{dT}\alpha_{2}^{(0)} + \frac{{dL_{4}^{(0)} }}{dT}, \\ Q_{31} (T) = & - 2\lambda_{2} (\lambda_{2} \rho_{0} + L_{6}^{(0)} )\alpha_{2}^{(0)} - (\lambda_{1} + \lambda_{2} )L_{5}^{(0)} p_{2}^{(0)} + \lambda_{2} L_{8}^{(0)} + \frac{{dL_{6}^{(0)} }}{dT}\beta_{2}^{(0)} + \frac{{dL_{5}^{(0)} }}{dT}q_{2}^{(0)} + \frac{{dL_{7}^{(0)} }}{dT}, \\ Q_{41} (T) = & \xi \ell^{\prime}_{2} r_{10} (2\lambda_{2} \rho_{0} + L_{6}^{(0)} ) - 2\lambda_{2} (\lambda_{2} \rho_{0} + L_{6}^{(0)} )\beta_{2}^{(0)} - L_{5}^{(0)} [(\lambda_{1} + \lambda_{2} )q_{2}^{(0)} + \Omega \ell^{\prime}_{2} r_{10} ] \\ & - \lambda_{2} L_{7}^{(0)} - \frac{{dL_{6}^{(0)} }}{dT}\alpha_{2}^{(0)} - \frac{{dL_{5}^{(0)} }}{dT}p_{2}^{(0)} + \frac{{dL_{8}^{(0)} }}{dT}. \\ \end{aligned}$$

Based on the $Q_{j1} (u)$ expressions, the solutions of the aforementioned system (22) may be formulated as follows

23 $$C_{j}^{(1)} (T) = \lambda_{k}^{ - 1} \int\limits_{0}^{T} {Q_{j1} (u)\sin } \lambda_{k} (T - u)du;\,\,\,\,\,\,k = \left\{ \begin{gathered} 1\,\,\,\,\,at\,\,j = 1,2 \hfill \\ 2\,\,\,\,at\,\,j = 3,4 \hfill \\ \end{gathered} \right.,$$

where $\lambda_{k}^{ - 1} Q_{j1} (T)$ can be obtained in terms of trigonometric functions, see (Appendix 2).

Substituting of $Q_{j1} (u)$ into (23), yields

24 $$\begin{aligned} C_{1}^{(1)} (T_{0} ) = & (E_{11} + R_{11} )T_{0} , \\ C_{2}^{(1)} (T_{0} ) = & (E_{21} + R_{21} )T_{0} , \\ C_{3}^{(1)} (T_{0} ) = & (E_{31} + R_{31} )T_{0} , \\ C_{4}^{(1)} (T_{0} ) = & (E_{41} + R_{41} )T_{0} , \\ \end{aligned}$$

where

25 $$\begin{aligned} E_{11} = & \frac{1}{2}\left[ {M_{2} (N_{19} - N_{110} ) - M_{1} N_{17} } \right], \\ E_{21} = & - \frac{1}{2}\left[ {M_{1} (N_{27} + N_{28} ) + M_{2} N_{210} } \right], \\ E_{31} = & - \frac{1}{2}M_{3} N_{51} , \\ E_{41} = & - \frac{1}{2}M_{3} N_{41} . \\ \end{aligned}$$

It goes without saying that we may express the necessary and sufficient criteria for $T$ periodic solutions of (15) as follows^33^

26 $$\psi_{j} = \Lambda_{2} (T_{0} ,\varepsilon ) - \Lambda_{2} (0,\varepsilon ) = 0;\,\,\,(j = 1,2,3,4),\,\,\,\,\,(\Lambda = p,q,\alpha ,\beta ).$$

Here, $\psi_{j}$ are expressed regarding to $M_{i} (i = 1,2,3),\,\,\rho ,$ and $\varepsilon$. These criteria determine $M_{i}^{(0)} ,\rho_{0} ,$ and $m_{j}$, and they are not independent according to system (11)^25^. In the scenario where $M_{3} \ne 0$, we may expect the third condition to directly result from the preceding conditions. Therefore, it is convenient to consider $M_{i}^{(0)}$ or $\rho_{0}$ as a constant, and one of $m_{j}$ as a function of *ε*^26^.

The below necessary criteria for achieving the periodicity of $C_{j}^{(1)}$ can be derived by dividing Eq. (26) by $\varepsilon$

27 $$C_{j}^{(1)} (T_{0} ) = C_{j}^{(1)} (M_{1} ,M_{2} ,M_{3} ,\varepsilon ) = 0.$$

To enhance our understanding of the aforementioned criteria, one can utilize Eq. (23) to get

28 $$\begin{gathered} E_{11} + R_{11} = 0, \hfill \\ E_{21} + R_{21} = 0, \hfill \\ E_{31} + R_{31} = 0, \hfill \\ E_{41} + R_{41} = 0. \hfill \\ \end{gathered}$$

It is clear that when the quotient of $\lambda_{1}$ divided by $\lambda_{2}$ equals $2$, $1/2$, $1$ or $- 1$, we get the following forms nonzero mathematical formulae of $R_{11}$, $R_{21}$, $R_{31}$ and $R_{41}$

29 $$\begin{gathered} i)\;for\;\lambda_{1} \lambda_{2}^{ - 1} = 2, \hfill \\ \quad R_{11} = - \frac{1}{2}N_{15} , \hfill \\ \,\,\,\,\,R_{21} = - \frac{1}{2}N_{25} , \hfill \\ \quad R_{31} = - \frac{1}{2}\left[ {M_{1} M_{3} (N_{311} + N_{312} ) + M_{2} M_{3} (N_{314} - N_{313} )} \right], \hfill \\ \quad R_{41} = - \frac{1}{2}\left[ {M_{1} M_{3} (N_{411} + N_{412} ) + M_{2} M_{3} (N_{414} - N_{413} )} \right]. \hfill \\ \end{gathered}$$

30 $$\begin{gathered} ii)\;for\;\lambda_{1} \lambda_{2}^{ - 1} = \frac{1}{2}, \hfill \\ \quad R_{11} = \frac{1}{2}[M_{1} M_{3} (N_{111} + N_{112} ) + M_{2} M_{3} (N_{114} - N_{113} )], \hfill \\ \quad R_{21} = \frac{1}{2}[M_{1} M_{3} (N_{211} + N_{212} ) + M_{2} M_{3} (N_{214} - N_{213} )], \hfill \\ \quad R_{31} = - \frac{1}{2}N_{33} ,\quad \,\,\,\,\,\,\,\,\,\,\,\, \hfill \\ \,\,\,\,\,R_{41} = - \frac{1}{2}N_{43} . \hfill \\ \end{gathered}$$

31 $$\begin{gathered} iii)\;for\;\lambda_{1} \lambda_{2}^{ - 1} = 1, \hfill \\ \quad R_{11} = \frac{1}{2}[M_{2} M_{3}^{2} (N_{122} - N_{121} ) - N_{123} - M_{3} N_{11} ], \hfill \\ \quad R_{21} = \frac{1}{2}[M_{1} M_{3}^{2} (N_{219} + N_{220} ) - M_{3} N_{21} - N_{223} ], \hfill \\ \quad R_{31} = - \frac{1}{2}[M_{1} N_{37} - M_{2} (N_{39} - N_{310} ) - M_{2} M_{3}^{2} (N_{322} - N_{321} ) + N_{323} ], \hfill \\ \quad R_{41} = - \frac{1}{2}[M_{2} N_{410} + M_{1} (N_{47} + N_{48} ) - M_{1} M_{3}^{2} (N_{419} + N_{420} ) + N_{423} ]. \hfill \\ \end{gathered}$$

32 $$\begin{gathered} iv)\;for\;\lambda_{1} \lambda_{2}^{ - 1} = - 1, \hfill \\ \quad R_{11} = \frac{1}{2}M_{3} N_{11} ,\,\,\,\,\,\,\,\,\,\,\,\,\,\,\quad \hfill \\ \,\,\,\,\,R_{21} = \frac{1}{2}M_{3} N_{21} , \hfill \\ \quad R_{31} = - \frac{1}{2}(M_{1} N_{37} + M_{2} N_{310} - M_{2} N_{39} ), \hfill \\ \quad R_{41} = \frac{1}{2}(M_{1} N_{47} + M_{1} N_{48} + M_{2} N_{410} ), \hfill \\ \end{gathered}$$

where $N_{1\Delta } ,N_{2\hbar } ,N_{3\Delta } ,$ and $N_{4\Delta } ;\,\,(\Delta = 1,2, \cdots ,24),\,\,\,(\hbar = 1,2, \cdots ,14)$ are given in (Appendix 3).

If $M_{i}^{(0)}$ and $\rho_{0}$ meet Eq. (28), then it is assumed that the Jacobi matrices of $C_{j}^{(1)} (T_{0} )$ are expressed in relation to $M_{i}$. Next, each of $\rho$ for $M_{i} = M_{i}^{(0)} ,\,\,\rho = \rho_{0} ,$ and $\psi_{j}$ must be determined in terms of *m*_*j*_, under the condition $m_{j} = \varepsilon = 0$**.** Since the calculation of the second matrix does not depend on *ε*, we can safely set *ε* = 0. As both *M*_*j*_, *ρ *and *m*_*j*_ are evident in the solutions as linked units, these matrices can be denoted by *J*. This suggests that these variables play a role in the outcomes or results being discussed, indicating their interconnectedness or dependence within the context of the problem or analysis. Hence, the solutions of Eq. (26) enable us to explore the necessary periodic solutions based on the following scenario.

## Irrational frequency scenario

This section elucidates the desired solutions pertaining to the scenario where $\lambda_{1} \lambda_{2}^{ - 1}$ is irrational. It is worth noting that one may derive these solutions when $M_{1}^{(0)} = M_{2}^{(0)} = E_{21} = E_{31} = 0$, $E_{11} \ne 0$, $M_{3}^{(0)} S_{53} \ne 0$ (for any quantity of $M_{3}$)^28^, *J* is from a third-rank, and

33 $$\begin{aligned} \rho_{0} = & \frac{1}{{2\lambda_{2} }}\left[ {S_{85} + S_{78} - 2S_{61} - 2M_{3}^{(0)2} S_{63} - \left( {2S_{62} + \frac{{\lambda_{1} }}{{\lambda_{2} }}S_{56} } \right)\left( {m_{1}^{2} + m_{2}^{2} } \right)} \right. \\ & \left. { + \frac{{\xi \ell^{\prime}_{2} r_{10} }}{{\lambda_{2} }}S_{68} - \frac{{\Omega \ell^{\prime}_{2} r_{10} }}{{\lambda_{1} }}S_{58} } \right], \\ \end{aligned}$$

are met.

In the current context, it is foreseeable that the solutions of Eq. (26) take the form of a power series in terms of the small parameter, involving $m_{1} ,m_{2} ,$ and $m_{4}$. Within this framework, we may assume that $m_{3}$ is negligible. Hence, these solutions become null at $\varepsilon = 0$. Building upon the outlined procedure, we can express the periodicity criteria as follows

34 $$\begin{gathered} m_{1} (\cos \lambda_{1} T_{0} - 1) + m_{2} \sin \lambda_{1} T_{0} + \varepsilon C_{1}^{(1)} (T_{0} ) + \ldots = 0, \hfill \\ - m_{1} \sin \lambda_{1} T_{0} + m_{2} (\cos \lambda_{1} T_{0} - 1) + \varepsilon C_{2}^{(1)} (T_{0} ) + \ldots = 0, \hfill \\ - (M_{3}^{(0)} + m_{3} )\sin \lambda_{2} T_{0} + C_{4}^{(1)} (T_{0} ) + \ldots = 0. \hfill \\ \end{gathered}$$

Based on the Eq. (30), we observe that the solutions $p_{2} (T,\varepsilon )$, $q_{2} (T,\varepsilon )$, $\alpha_{2} (T,\varepsilon ),$ and $\beta_{2} (T,\varepsilon )$ of the unperturbed scenario ($\varepsilon = 0$) have the following forms

35 $$\begin{aligned} p_{2} = & M_{1}^{(0)} \cos \lambda_{1} T + M_{2}^{(0)} \sin \lambda_{1} T, \\ q_{2} = & - M_{1}^{(0)} \sin \lambda_{1} T + M_{2}^{(0)} \cos \lambda_{1} T - \frac{{\Omega \ell^{\prime}_{2} r_{10} }}{{\lambda_{1} }}, \\ \alpha_{2} = & M_{3}^{(0)} \cos \lambda_{1} T, \\ \beta_{2} = & - M_{3}^{(0)} \sin \lambda_{2} T + \frac{{\xi \ell^{\prime}_{2} r_{10} }}{{\lambda_{2} }}. \\ \end{aligned}$$

Using (32), one obtains directly $m_{1}$ and $m_{2}$ as follows

36 $$\begin{aligned} m_{1} = & \varepsilon \left\{ {\left\{ {\frac{1}{4}M_{3}^{(0)} \left[ {S_{48} + S_{35} - \frac{{\Omega \ell^{\prime}_{2} r_{10} }}{{\lambda_{1} }}S_{15} } \right]T_{0} \cot \frac{{\lambda_{1} }}{2}T} \right._{0} } \right. \\ & \left. { + \frac{1}{4}M_{3}^{(0)} [S_{48} + S_{35} - \frac{{\xi \ell^{\prime}_{2} r_{10} }}{{\lambda_{1} }}S_{25} - \frac{{\Omega \ell^{\prime}_{2} r_{10} }}{{\lambda_{1} }}S_{15} ]T_{0} } \right\} + ..., \\ m_{2} = & \varepsilon \left\{ {\frac{1}{4}M_{3}^{(0)} \left[ {S_{48} + S_{35} - \frac{{\xi \ell^{\prime}_{2} r_{10} }}{{\lambda_{1} }}S_{25} - \frac{{\Omega \ell^{\prime}_{2} r_{10} }}{{\lambda_{1} }}S_{15} } \right]T_{0} \cot \frac{{\lambda_{1} }}{2}T_{0} } \right. \\ & \left. { + \frac{1}{4}M_{3}^{(0)} \left[ {S_{48} + S_{35} - \frac{{(2 + \xi h)\ell^{\prime}_{2} r_{10} }}{{\lambda_{1} }}S_{15} } \right]} \right\} + \ldots \\ \end{aligned}$$

In terms of power series of $\varepsilon$ and based on the aforementioned formulas in (4), (7), (10), (15), (23), and (24), one may achieve the required solutions in the form

$$\begin{aligned} p = & \varepsilon^{1/2} n^{ - 1} \left( {c_{1} + M_{3}^{(0)} h\cos \lambda_{2} T} \right) + \varepsilon^{3/2} n^{ - 1} \left\{ {\frac{1}{4}M_{3}^{(0)} \left[ {S_{48} + S_{35} - \frac{{(2 + \xi h)\ell^{\prime}_{2} r_{10} }}{{\lambda_{1} }} \times S_{15} } \right]T_{0} \cot \frac{{\lambda_{1} }}{2}T_{0} }  + \frac{1}{4}M_{3}^{(0)} \left[ {S_{48} + S_{35} - \frac{{\xi \ell^{\prime}_{2} r_{10} }}{{\lambda_{1} }}S_{25} - \frac{{\Omega \ell^{\prime}_{2} r_{10} }}{{\lambda_{1} }}S_{15} } \right]T_{0} \right\}\cos \lambda_{1} T \\ & + \varepsilon^{3/2} n^{ - 1} \left\{ {\frac{1}{4}M_{3}^{(0)} \left[ {S_{48} + S_{35} - \frac{{\xi \ell^{\prime}_{2} r_{10} }}{{\lambda_{1} }}S_{25} - \frac{{\Omega \ell^{\prime}_{2} r_{10} }}{{\lambda_{1} }}S_{15} } \right]T_{0} \cot \frac{{\lambda_{1} }}{2}T_{0} + \frac{1}{4}M_{3}^{(0)} } \right. \\ & \left. { \times \left[ {S_{48} + S_{35} - \frac{{(2 + \xi h)\ell^{\prime}_{2} r_{10} }}{{\lambda_{1} }}S_{15} } \right]T_{0} } \right\}\sin \lambda_{1} T + \varepsilon^{3/2} n^{ - 1} \left\{ {\frac{1}{{\lambda_{1} }}[S_{41} + \left( {S_{43} - \frac{1}{2}S_{25} } \right)} \right. \\ & \left. { \times M_{3}^{(0)2} } \right](1 - \cos \lambda_{1} T) - \frac{1}{2}\left[ {S_{48} + S_{35} - \frac{{\xi \ell^{\prime}_{2} r_{10} }}{{\lambda_{1} }}S_{25} - \frac{{\Omega \ell^{\prime}_{2} r_{10} }}{{\lambda_{1} }}S_{15} } \right]M_{3}^{(0)} \left( {T\cos \lambda_{1} T - \frac{1}{{\lambda_{1} }}\sin \lambda_{1} T} \right) \\ & + \frac{1}{2}\left[ {S_{45} - S_{38} + \frac{{\xi \ell^{\prime}_{2} r_{10} }}{{\lambda_{1} }}S_{28} + \frac{{\Omega \ell^{\prime}_{2} r_{10} }}{{\lambda_{1} }}S_{18} } \right]M_{3}^{(0)} T\sin \lambda_{1} T - M_{3}^{(0)2} \\ & \left. { \times S_{28} \left( {\frac{1}{{6\lambda_{1} }}\sin 2\lambda_{1} T - \frac{1}{{3\lambda_{1} }}\sin \lambda_{1} T} \right) - M_{3}^{(0)2} S_{25} \left( {\frac{1}{{6\lambda_{1} }}\cos 2\lambda_{1} T - \frac{1}{{6\lambda_{1} }}\cos \lambda_{1} T} \right)} \right\} \\ & + \varepsilon^{3/2} n^{ - 1} h\{ \frac{1}{{\lambda_{2} }}\left[ {S_{81} + (S_{83} - \frac{1}{2}S_{65} )M_{3}^{(0)2} } \right](1 - \cos \lambda_{2} T) + \frac{1}{2}\left[ {S_{88} - S_{75} - \frac{{\xi \ell^{\prime}_{2} r_{10} }}{{\lambda_{2} }}} \right. \\ & \left. { \times S_{65} - \frac{{\Omega \ell^{\prime}_{2} r_{10} }}{{\lambda_{2} }}S_{55} } \right]M_{3}^{(0)} \left( {T\cos \lambda_{2} T - \frac{1}{{\lambda_{2} }}\sin \lambda_{2} T} \right) - M_{3}^{(0)2} S_{68} \left( {\frac{1}{{2\lambda_{2} }}\sin 2\lambda_{2} T - \frac{1}{{\lambda_{2} }}\sin \lambda_{2} T} \right) \\ & \left. { - M_{3}^{(0)2} S_{65} \left( {\frac{1}{{2\lambda_{2} }}\cos 2\lambda_{2} T - \frac{1}{{2\lambda_{2} }}\cos \lambda_{2} T} \right)} \right\} + \ldots , \\ \end{aligned}$$

$$\begin{aligned} q = & - \varepsilon^{1/2} n^{ - 1} \left[ {\frac{{\Omega \ell^{\prime}_{2} r_{10} }}{{\lambda_{1} }} - \varepsilon^{3/2} n^{ - 1} \left\{ {\frac{1}{4}M_{3}^{(0)} \left[ {S_{48} + S_{35} - \frac{{(2 + \xi h)\ell^{\prime}_{2} r_{10} }}{{\lambda_{1} }}S_{15} } \right]T_{0} \cot \frac{{\lambda_{1} }}{2}T_{0} } \right.} \right. \\ & \left. { + \frac{1}{4}M_{3}^{(0)} \left[ {S_{48} + S_{35} - \frac{{\xi \ell^{\prime}_{2} r_{10} }}{{\lambda_{1} }}S_{25} - \frac{{\Omega \ell^{\prime}_{2} r_{10} }}{{\lambda_{1} }}S_{15} } \right]T_{0} } \right\}\sin \lambda_{1} T + \varepsilon^{3/2} n^{ - 1} \left\{ {\frac{1}{4}M_{3}^{(0)} [S_{48} } \right. \\ & \left. {\left. { + S_{35} - \frac{{\xi \ell^{\prime}_{2} r_{10} }}{{\lambda_{1} }}S_{25} - \frac{{\Omega \ell^{\prime}_{2} r_{10} }}{{\lambda_{1} }}S_{15} } \right]T_{0} \cot \frac{{\lambda_{1} }}{2}T_{0} + \frac{1}{4}M_{3}^{(0)} \left[ { - \frac{{(2 + \xi h)\ell^{\prime}_{2} r_{10} }}{{\lambda_{1} }}S_{15} + S_{48} + S_{35} } \right]T_{0} } \right\}\cos \lambda_{1} T \\ & + \varepsilon^{3/2} n^{ - 1} \left\{ {\frac{1}{{\lambda_{1} }}[(S_{11} + M_{3}^{(0)2} S_{13} )\frac{{(2 + \xi h)\ell^{\prime}_{2} r_{10} }}{{\lambda_{1} }} - S_{51} - (S_{33} - \frac{1}{2}S_{28} )} \right. \\ & \left. { \times M_{3}^{(0)2} - \frac{1}{{\lambda_{1} }}\Omega \ell^{\prime}_{2} r_{10} \left[ {S_{85} + S_{78} - 2(S_{61} + M_{3}^{(0)2} S_{63} ) - \frac{{\Omega \ell^{\prime}_{2} r_{10} }}{{\lambda_{1} }}S_{58} - \frac{{\xi \ell^{\prime}_{2} r_{10} }}{{\lambda_{1} }}S_{68} } \right]} \right] \\ & \times (1 - \cos \lambda_{1} T) - \frac{1}{2}\left[ {S_{45} - S_{38} + \frac{{(2 + \xi h)\ell^{\prime}_{2} r_{10} }}{{\lambda_{1} }}S_{18} } \right]M_{3}^{(0)} (T\cos \lambda_{1} T - \frac{1}{{\lambda_{1} }}\sin \lambda_{1} T) \\ & - \frac{1}{2}[S_{48} + S_{35} + \frac{{(2 + \xi h)\ell^{\prime}_{2} r_{10} }}{{\lambda_{1} }}S_{18} ]M_{3}^{(0)} T\sin \lambda_{1} T + M_{3}^{(0)2} S_{25} (\frac{1}{{6\lambda_{1} }}\sin 2\lambda_{1} T - \frac{1}{{3\lambda_{1} }} \times \sin \lambda_{1} T) \\ & \left. { + M_{3}^{(0)2} S_{28} (\frac{1}{{6\lambda_{1} }}\cos 2\lambda_{1} T - \frac{1}{{6\lambda_{1} }}\cos \lambda_{1} T)} \right\} + \varepsilon^{1/2} n^{ - 1} h(\frac{{\xi \ell^{\prime}_{2} r_{10} }}{{\lambda_{2} }} - M_{3}^{(0)} \times \sin \lambda_{2} T) \\ & + \varepsilon^{3/2} n^{ - 1} h\left\{ {\frac{1}{{\lambda_{2} }}[(S_{51} + M_{3}^{(0)2} S_{53} )\frac{{\Omega \ell^{\prime}_{2} r_{10} }}{{\lambda_{2} }} - (S_{61} + M_{3}^{(0)2} S_{63} )\frac{{\xi \ell^{\prime}_{2} r_{10} }}{{\lambda_{2} }}} \right. \\ & - (S_{71} + M_{3}^{(0)2} S_{73} ) + \frac{3}{2}M_{3}^{(0)2} S_{68} ](1 - \cos \lambda_{2} T) + \frac{1}{2}[S_{88} - S_{75} - \frac{{\xi \ell^{\prime}_{2} r_{10} }}{{\lambda_{2} }}S_{65} \\ & - \frac{{\Omega \ell^{\prime}_{2} r_{10} }}{{\lambda_{2} }}S_{55} ]M_{3}^{(0)} T\sin \lambda_{2} T + M_{3}^{(0)2} S_{65} (\frac{1}{{2\lambda_{2} }}\sin 2\lambda_{2} T - \frac{1}{{\lambda_{2} }}\sin \lambda_{2} T) + M_{3}^{(0)2} \\ & \times S_{68} (\frac{1}{{6\lambda_{2} }}\cos 2\lambda_{2} T - \frac{1}{{6\lambda_{2} }}\cos \lambda_{2} T) + ....\,\,, \\ \end{aligned}$$

$$\begin{aligned} r = & n^{ - 1} r_{10} - \frac{{\varepsilon n^{ - 1} }}{{br_{10} }}\left\{ {[h(d_{1} - \xi ) - 1 + d_{2} (1 - \xi h)]M_{3}^{(0)} (\cos \lambda_{2} T - 1)} \right\} - \varepsilon n^{ - 1} \frac{{\ell^{\prime}_{2} }}{{b\lambda_{1} \lambda_{2} }} \\ & \times \left\{ {h[\xi h(\lambda_{2} - \lambda_{1} ) + \lambda_{2} ] + \xi (\lambda_{2} - \lambda_{1} )(1 - \xi h)^{2} ]} \right\}M_{3}^{(0)} \sin \lambda_{2} T + \ldots , \\ \end{aligned}$$

$$\begin{gathered} \alpha = \varepsilon^{1/2} [c_{2} + \Omega M_{3}^{(0)} \cos \lambda_{2} T] + \varepsilon^{3/2} \Omega \{ \frac{1}{{\lambda_{2} }}[S_{81} + (S_{83} + \frac{1}{2}S_{13} )M_{3}^{(0)2} (1 - \cos \lambda_{2} T) \hfill \\ \quad \; + \frac{1}{2}M_{3}^{(0)} [S_{88} - S_{75} - \frac{{\xi \ell^{\prime}_{2} r_{10} }}{{\lambda_{2} }}S_{65} - \frac{{\Omega \ell^{\prime}_{2} r_{10} }}{{\lambda_{2} }}S_{55} ](T\cos \lambda_{2} T - \frac{1}{{\lambda_{2} }}\sin \lambda_{2} T) - M_{3}^{(0)2} \hfill \\ \quad \; \times S_{68} (\frac{1}{{2\lambda_{2} }}\sin 2\lambda_{2} T - \frac{1}{{\lambda_{2} }}\sin \lambda_{2} T) - M_{3}^{(0)2} S_{65} (\frac{1}{{2\lambda_{2} }}\cos 2\lambda_{2} T - \frac{1}{{2\lambda_{2} }}\cos \lambda_{2} T)\} \hfill \\ \quad \; + \varepsilon^{3/2} \xi \{ \frac{1}{4}M_{3}^{(0)} [S_{48} + S_{35} - \frac{{(2 + \xi h)\ell^{\prime}_{2} r_{10} }}{{\lambda_{1} }}S_{15} ]T_{0} \cot \frac{{\lambda_{1} }}{2}T_{0} + \frac{1}{4}M_{3}^{(0)} [S_{48} + S_{35} \hfill \\ \quad \; - \frac{{\xi \ell^{\prime}_{2} r_{10} }}{{\lambda_{1} }}S_{25} - \frac{{\Omega \ell^{\prime}_{2} r_{10} }}{{\lambda_{1} }}S_{15} ]T_{0} \} \cos \lambda_{1} T + \varepsilon^{3/2} \xi \{ \frac{1}{4}M_{3}^{(0)} \{ [S_{48} + S_{35} - \frac{{\xi \ell^{\prime}_{2} r_{10} }}{{\lambda_{1} }}S_{25} \hfill \\ \quad \; - \frac{{\Omega \ell^{\prime}_{2} r_{10} }}{{\lambda_{1} }}S_{15} ]T_{0} \cot \frac{{\lambda_{1} }}{2}T_{0} + \frac{1}{4}M_{3}^{(0)} [S_{48} + S_{35} - \frac{{(2 + \xi h)\ell^{\prime}_{2} r_{10} }}{{\lambda_{1} }}S_{15} ]\} \sin \lambda_{1} T \hfill \\ \quad \; + \varepsilon^{3/2} \xi \{ \frac{1}{{\lambda_{1} }}[S_{41} + (S_{43} + \frac{1}{2}S_{25} )M_{3}^{(0)2} ](1 - \cos \lambda_{1} T) - \frac{1}{2}M_{3}^{(0)} [S_{48} + S_{35} - \frac{{\xi \ell^{\prime}_{2} r_{10} }}{{\lambda_{1} }} \hfill \\ \quad \; \times S_{25} - \frac{{\Omega \ell^{\prime}_{2} r_{10} }}{{\lambda_{1} }}S_{15} ](T\cos \lambda_{1} T - \frac{1}{{\lambda_{1} }}\sin \lambda_{1} T) + \frac{1}{2}M_{3}^{(0)} [S_{45} - S_{38} + \frac{{\xi \ell^{\prime}_{2} r_{10} }}{{\lambda_{1} }}S_{28} \hfill \\ \quad \; + \frac{{\Omega \ell^{\prime}_{2} r_{10} }}{{\lambda_{1} }}S_{18} ]T\sin \lambda_{1} T - M_{3}^{(0)2} S_{28} (\frac{1}{{6\lambda_{1} }}\sin 2\lambda_{1} T - \frac{1}{{3\lambda_{1} }}\sin \lambda_{1} T) - M_{3}^{(0)2} S_{25} \hfill \\ \quad \; \times (\frac{1}{{6\lambda_{1} }}\cos 2\lambda_{1} T - \frac{1}{{6\lambda_{1} }}\cos \lambda_{1} T)\} + .... \hfill \\ \end{gathered}$$

$$\begin{aligned} \beta = & \varepsilon^{1/2} \Omega \left( {\frac{{\xi \ell^{\prime}_{2} r_{10} }}{{\lambda_{2} }} - M_{3}^{(0)} \sin \lambda_{2} T} \right) + \varepsilon^{3/2} \Omega \left\{ {\frac{1}{{\lambda_{2} }}[\left( {S_{51} + M_{3}^{(0)2} S_{53} } \right)\frac{{\Omega \ell^{\prime}_{2} r_{10} }}{{\lambda_{2} }}} \right. \\ & - [S_{61} + M_{3}^{(0)2} S_{63} ]\frac{{\xi \ell^{\prime}_{2} r_{10} }}{{\lambda_{2} }} - [S_{71} + M_{3}^{(0)2} S_{73} ] + \frac{3}{2}M_{3}^{(0)2} S_{68} ](1 - \cos \lambda_{2} T) + \frac{1}{2}M_{3}^{(0)} \\ & \times [S_{88} - S_{75} - \frac{{\xi \ell^{\prime}_{2} r_{10} }}{{\lambda_{1} }}S_{65} - \frac{{\Omega \ell^{\prime}_{2} r_{10} }}{{\lambda_{1} }}S_{55} ]T\sin \lambda_{2} T + M_{3}^{(0)2} S_{65} (\frac{1}{{2\lambda_{2} }}\sin 2\lambda_{2} T \\ & - \frac{1}{{\lambda_{2} }}\sin \lambda_{2} T) + M_{3}^{(0)2} S_{68} (\frac{1}{{2\lambda_{2} }}\cos 2\lambda_{2} T - \frac{1}{{2\lambda_{2} }}\cos \lambda_{2} T)\} - \varepsilon^{1/2} \xi \frac{{\Omega \ell^{\prime}_{2} r_{10} }}{{\lambda_{1} }} \\ & - \varepsilon^{3/2} \xi \{ \frac{1}{4}M_{3}^{(0)} [S_{48} + S_{35} - \frac{{(2 + \xi h)\ell^{\prime}_{2} r_{10} }}{{\lambda_{1} }}S_{15} ]T_{0} \cot \frac{{\lambda_{1} }}{2}T_{0} + \frac{1}{4}M_{3}^{(0)} [S_{48} + S_{35} \\ & - \frac{{\xi \ell^{\prime}_{2} r_{10} }}{{\lambda_{1} }}S_{25} - \frac{{\Omega \ell^{\prime}_{2} r_{10} }}{{\lambda_{1} }}S_{15} ]T_{0} \} \sin \lambda_{1} T + \varepsilon^{3/2} \xi \left\{ {\frac{1}{4}M_{3}^{(0)} [S_{48} + S_{35} - \frac{{\xi \ell^{\prime}_{2} r_{10} }}{{\lambda_{1} }}S_{25} } \right. \\ & \left. { - \frac{{\Omega \ell^{\prime}_{2} r_{10} }}{{\lambda_{1} }}S_{15} ]T_{0} \cot \frac{{\lambda_{1} }}{2}T_{0} + \frac{1}{4}M_{3}^{(0)} [S_{48} + S_{35} - \frac{{(2 + \xi h)\ell^{\prime}_{2} r_{10} }}{{\lambda_{1} }}S_{15} ]T_{0} } \right\}\cos \lambda_{1} T \\ & + \varepsilon^{3/2} \xi \left\{ {\frac{1}{{\lambda_{1} }}[S_{11} + M_{3}^{(0)2} S_{13} ]\frac{{(2 + \xi h)\ell^{\prime}_{2} r_{10} }}{{\lambda_{1} }} - S_{31} - (S_{33} + \frac{1}{2}S_{28} )M_{3}^{(0)2} - \frac{{\Omega \ell^{\prime}_{2} r_{10} }}{{\lambda_{1} }}} \right. \\ & \times \left[ {S_{85} + S_{78} - 2(S_{61} + M_{3}^{(0)2} S_{63} ) - \frac{{\xi \ell^{\prime}_{2} r_{10} }}{{\lambda_{1} }}S_{68} - \frac{{\Omega \ell^{\prime}_{2} r_{10} }}{{\lambda_{1} }}S_{58} } \right](1 - \cos \lambda_{1} T) - \frac{1}{2} \\ & \times M_{3}^{(0)} [S_{45} - S_{38} + \frac{{(2 + \xi h)\ell^{\prime}_{2} r_{10} }}{{\lambda_{1} }}S_{18} ](T\cos \lambda_{1} T - \frac{1}{{\lambda_{1} }}\sin \lambda_{1} T) - \frac{1}{2}M_{3}^{(0)} [S_{48} + S_{35} \\ & - \frac{{(2 + \xi h)\ell^{\prime}_{2} r_{10} }}{{\lambda_{1} }}S_{15} ]T\sin \lambda_{1} T + M_{3}^{(0)2} S_{25} \left( {\frac{1}{{6\lambda_{1} }}\sin 2\lambda_{1} T - \frac{1}{{3\lambda_{1} }}\sin \lambda_{1} T} \right) \\ & \left. { + M_{3}^{(0)2} S_{28} \left( {\frac{1}{{6\lambda_{1} }}\cos 2\lambda_{1} T - \frac{1}{{6\lambda_{1} }}\cos \lambda_{1} T} \right)} \right\} + ..., \\ \end{aligned}$$

$$\begin{aligned} \gamma = & 1 - \varepsilon \left\{ {\frac{1}{2}[c_{2}^{2} + \Omega^{2} M_{3}^{(0)2} ] + c_{2} \Omega M_{3}^{(0)} \cos \lambda_{2} T + \sin \lambda_{2} TM_{3}^{(0)} \frac{{\Omega^{2} \xi \ell^{\prime}_{2} r_{10} (\lambda_{2} - \lambda_{1} )}}{{\lambda_{1} \lambda_{2} }}} \right. \\ & + \left. {\frac{1}{2}\left[ {\frac{{\Omega^{2} \xi \ell^{\prime}_{2} r_{10} (\lambda_{2} - \lambda_{1} )}}{{\lambda_{1} \lambda_{2} }}} \right]^{2} } \right\} + \cdots , \\ \end{aligned}$$

37 $$T_{0} = 2\pi \lambda_{1}^{ - 1} .$$

Drawing from the aforementioned solutions achieved in the system of Eq. (37), it can be inferred that they are formulated in relation to the small parameter $\varepsilon$, in addition to trigonometric functions. Therefore, it is reasonable to anticipate that these solutions will exhibit periodic or quasi-periodic behavior, in which some constants are observed, may influence the overall periodicity.

## Euler’s angles and explanation of motion

The aim of this section is to focus on obtaining approximate mathematical representations for the angles of Euler, specifically in relation to $\varepsilon$ within the context of the problem being studied. Leveraging the attained solutions (35), we aim to analyse the movement at any given instant. To accomplish this task, let us examine the below expressions for the angles $\theta ,\psi ,$ and $\varphi$^1,23^

38 $$\begin{aligned} \theta = & \cos^{ - 1} \gamma , \\ \dot{\psi } = & {{(p{\kern 1pt} {\kern 1pt} \alpha + q{\kern 1pt} {\kern 1pt} {\kern 1pt} \beta )} \mathord{\left/ {\vphantom {{(p{\kern 1pt} {\kern 1pt} \alpha + q{\kern 1pt} {\kern 1pt} {\kern 1pt} \beta )} {(1 - \gamma^{2} )}}} \right. \kern-0pt} {(1 - \gamma^{2} )}}, \\ \dot{\varphi } = & r - \dot{\psi }\cos \theta ,\,(. \equiv {d \mathord{\left/ {\vphantom {d {dt}}} \right. \kern-0pt} {dt}}\;). \\ \end{aligned}$$

Utilizing the aforementioned formulas in (36) alongside the acquired solutions in (35), we can achieve the following forms of $\theta ,\psi ,$ and $\varphi$

39 $$\begin{aligned} \theta = & \theta_{0} + \varepsilon c_{2} \Omega M_{3}^{(0)} \cos ec\theta_{0} \left[ {\cos \frac{{\lambda_{1} (t + h)}}{n} - \cos \frac{{\lambda_{1} t}}{n}} \right] + ..., \\ \psi = & \psi_{0} - \frac{\varepsilon }{n}\cos ec^{2} \theta_{0} \left\{ {\left[ {c_{1} c_{2} + h\Omega (M_{3}^{(0)} )^{2} } \right]t + \frac{{n\Omega \ell^{\prime}_{2} r_{10} }}{{\lambda_{1} }}\left( {\cos \frac{{\lambda_{1} t}}{n} - 1} \right)} \right. \\ & \left. { + [c_{2} h + c_{1} \Omega ]M_{3}^{(0)} \frac{n}{{\lambda_{1} }}\sin \frac{{\lambda_{1} t}}{n}} \right\} + ..., \\ \varphi = & \,\varphi_{0} + \frac{{r_{10} }}{n}t - \frac{\varepsilon }{{nbr_{10} }}M_{3}^{(0)} \left\{ {\left[ {(c_{1} - \xi )h - 1 - c_{2} } \right]\left( {\frac{n}{{\lambda_{1} }}\sin \frac{{\lambda_{1} t}}{n} - t} \right) + \frac{nh}{{\lambda_{1}^{2} }}\ell^{\prime}_{2} r_{10} } \right. \\ & \left. { \times \left( {\cos \frac{{\lambda_{1} t}}{n} - 1} \right)} \right\} - \frac{\varepsilon }{n}\cos {\text{ec}}^{2} \theta_{0} \cos \theta_{0} \left\{ {\left[ {c_{1} c_{2} + h\Omega (M_{3}^{(0)} )^{2} } \right]t + \frac{n}{{\lambda_{1}^{2} }}\Omega \ell^{\prime}_{2} r_{10} } \right. \\ & \left. { \times \left( {\cos \frac{{\lambda_{1} t}}{n} - 1} \right) + \frac{n}{{\lambda_{1} }}\left[ {c_{2} h + c_{1} \Omega } \right]M_{3}^{(0)} \sin \frac{{\lambda_{1} t}}{n}} \right\} + \ldots ; \\ \varphi_{0} = & \tan^{ - 1} ({{\alpha_{0} } \mathord{\left/ {\vphantom {{\alpha_{0} } {\beta_{0} }}} \right. \kern-0pt} {\beta_{0} }}), \\ \psi_{0} = & \frac{\pi }{2} - \frac{{\lambda_{1} h}}{n}. \\ \end{aligned}$$

It is evident that the above expressions (39) are contingent upon certain arbitrary constants denoted by $\theta_{0} ,\,\psi_{0} ,$ and $r_{0}$ of the respective variables.

## Interpretation of the outcomes

This section’s main goal is to simulate and examine the aforementioned outcomes in (37) and (39) through the following graphs in light of the positive impact of the system’s parameters. This presentation aims to underscore the importance of external applied forces and moments on the system’s behavior. To fulfil this objective, let us take into consideration the below data

40 $$\begin{gathered} A = B = 150\;{\text{kg}}\,{\text{m}}^{{2}} ,\;\;C = 130\,{\text{kg}}\,{\text{m}}^{{2}} ,\varepsilon = 0.1,\;\;x_{c} = 0.04\;{\text{m}},\;\;H = 100, \hfill \\ M = 100\,{\text{kg}},\quad g = 9.81\;{\text{m}}\,{\text{s}}^{ - 2} ,\;\;r_{0} = 0.1,\;\;\ell_{2} ,\ell_{3} ( = 10,30,50)\,{\text{kg}}\,{\text{m}}^{{2}} \,{\text{s}}^{ - 1} , \hfill \\ e( = 0.01,0.2,0.4){\text{C,}}\;\;\theta_{0} = 0.1\;{\text{rad}}. \hfill \\ \end{gathered}$$

**Figure 2:**
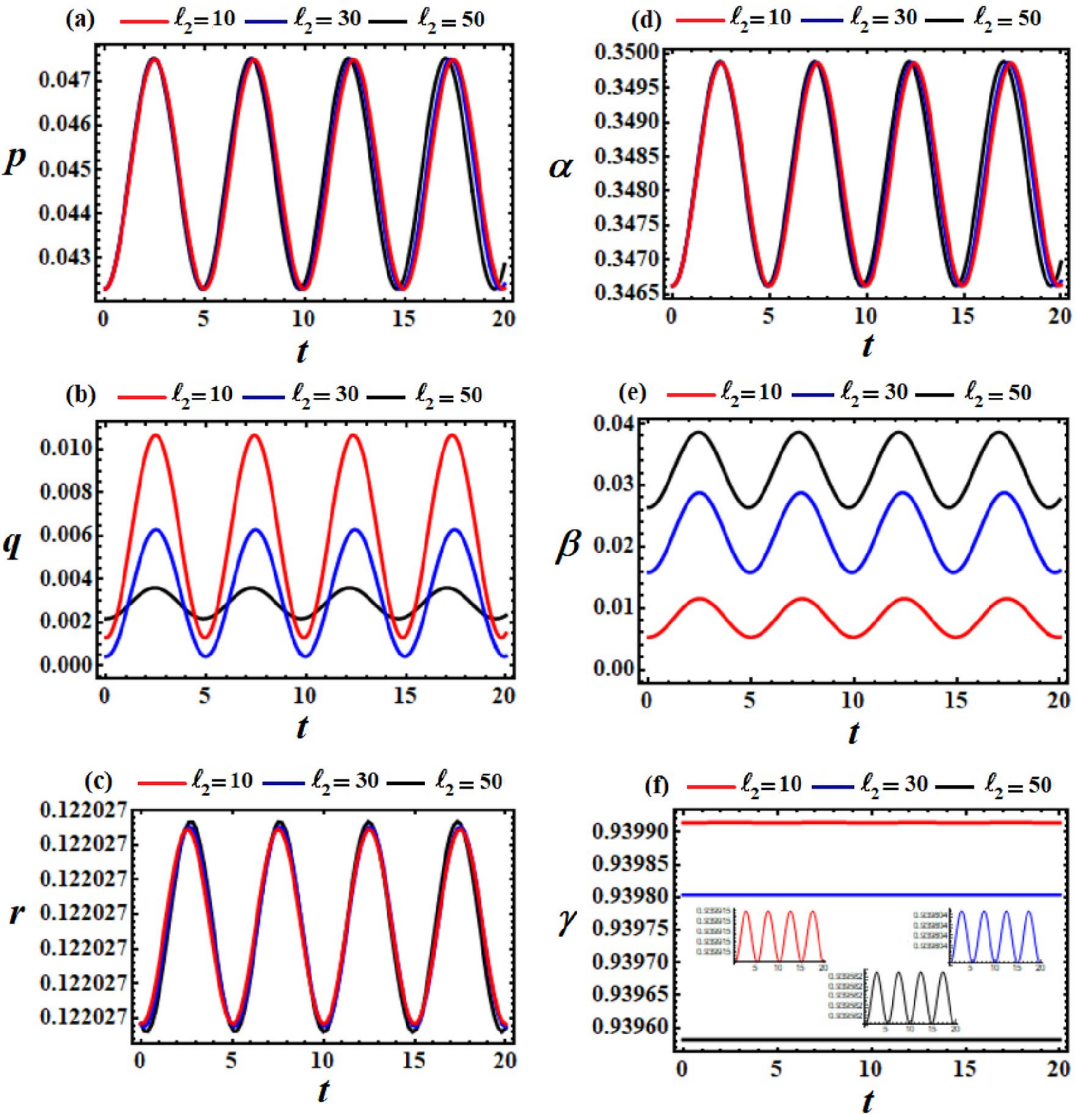
Displays the temporal histories of the gained solutions at $\ell_{3} = 10\,{\text{kg}}\,{\text{m}}^{2} \,{\text{s}}^{ - 1} ,$ $\ell_{2} = (10,30,50)\,{\text{kg}}\,{\text{m}}^{2} \,{\text{s}}^{ - 1} ,$ and $M = 100\,{\text{kg}}$.

**Figure 3:**
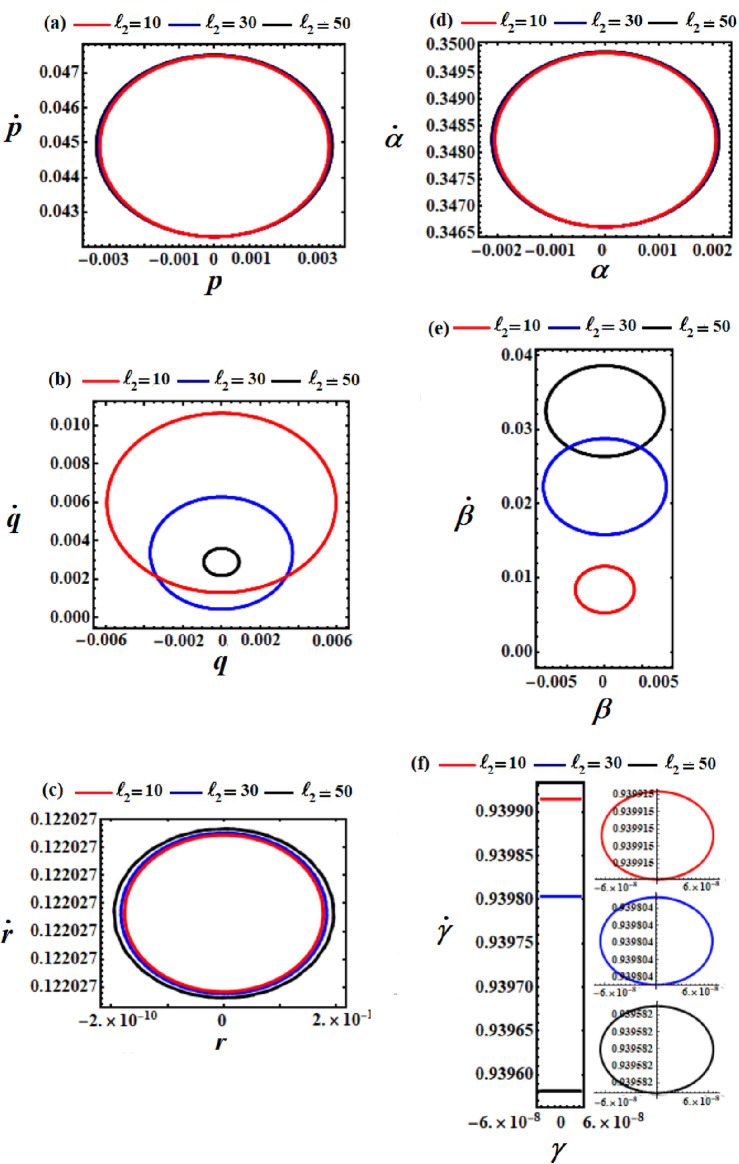
Displays the difference in solutions $p,q,r,\alpha ,\beta ,$ and $\gamma$ via *t* at $\ell_{2} = (10,30,50)\,{\text{kg}}\,{\text{m}}^{2} \,{\text{s}}^{ - 1} ,$ $\ell_{3} = 10\,{\text{kg}}\,{\text{m}}^{2} \,{\text{s}}^{ - 1} ,$ and $M = 100\,{\text{kg}}$.

**Figure 4:**
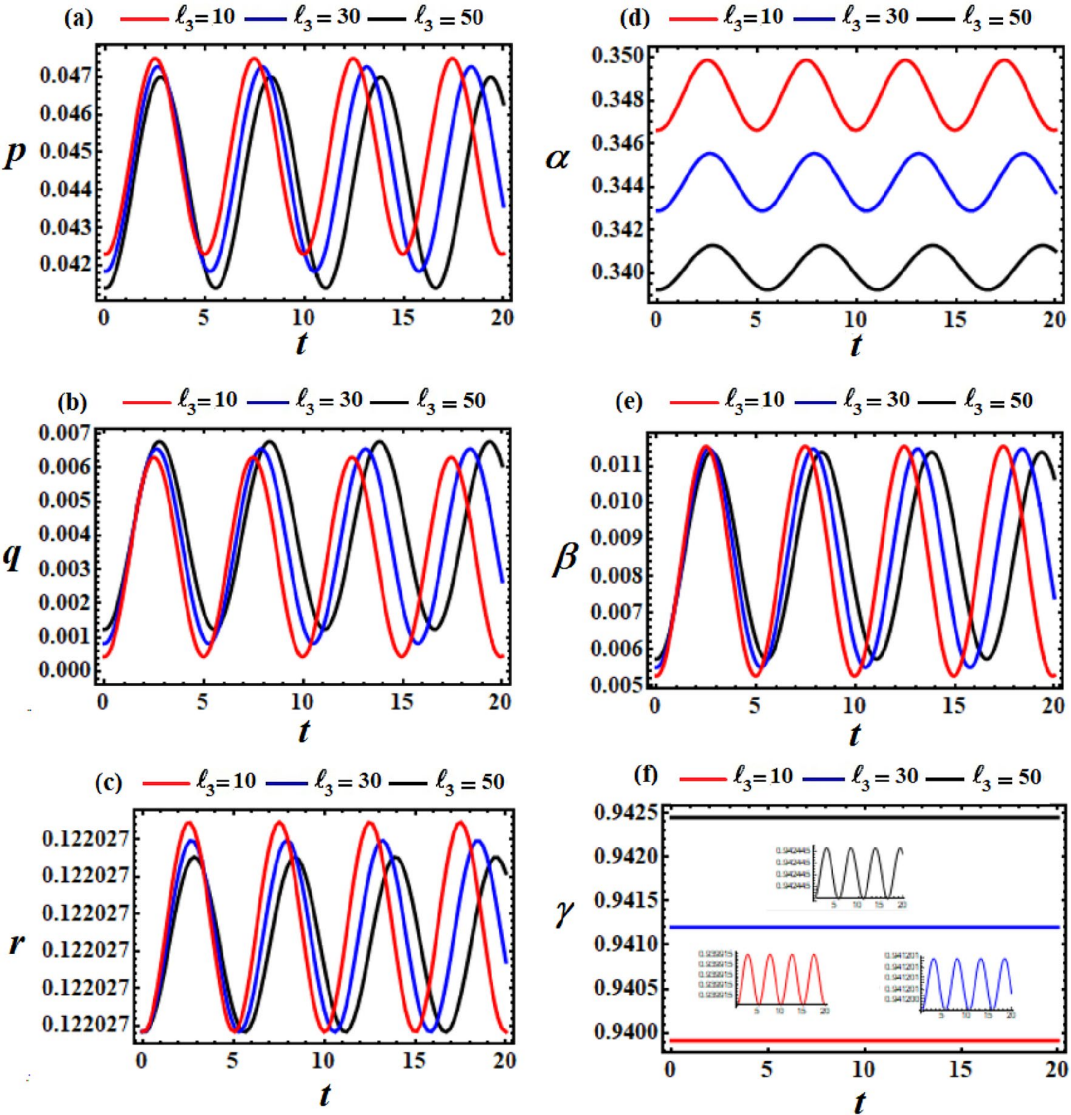
Displays the difference in the obtained solutions via *t* at $\ell_{3} = (10,30,50)\,{\text{kg}}\,{\text{m}}^{2} \,{\text{s}}^{ - 1} ,$ and $\ell_{2} = 10\,{\text{kg}}\,{\text{m}}^{2} \,{\text{s}}^{ - 1}$.

Figures 2, 4 and 6 present the temporal evolution of the achieved outcomes in the system of Eqs. (37), when the values of $\ell_{2} ,\ell_{3} ,$ and $e$ have various values, respectively. The corresponding diagrams of phase planes are demonstrated in Figs. 3, 4, and 7 for identical parameter settings. These illustrations are calculated based on the preceding data provided in (40).

The primary objective of the curves in Fig. 2 is to investigate the favorable impacts of the chosen values of the second component $\ell_{2} ( = 10,30,50)$ on the body’s behavior. It is noted that periodic waves are evident in the sections of this figure, as expected before. The solutions $p,r,$ and $\alpha$ exhibit slight variations with the various values of $\ell_{2}$, as explored in Figs. 2a, c, and d. This behavior can be attributed to the formulas governing these solutions, which either do not explicitly depend on $\ell_{2}$ or express it within a bracket alongside $\varepsilon$. Nevertheless, the remaining parts (b), (e), and (f) are notably affected by the change of $\ell_{2}$ values. It is observed that the waves’ amplitudes increase with an increase in $\ell_{2}$, as illustrated in Figs. 2e and f. Furthermore, neither the oscillations’ number nor the wave’s wavelengths vary.

The diagrams illustrating the body’s steady motion are drawn by phase plane plots for the achieved solutions above, when $\ell_{2} ( = 10,30,50)$, as seen in Fig. 3. These plots are generated for the solutions and their first derivative, in which time has been excluded. It is evident that symmetric closed curves are presented, serving to characterize and confirm the stability of the body’s behavior.

**Figure 5:**
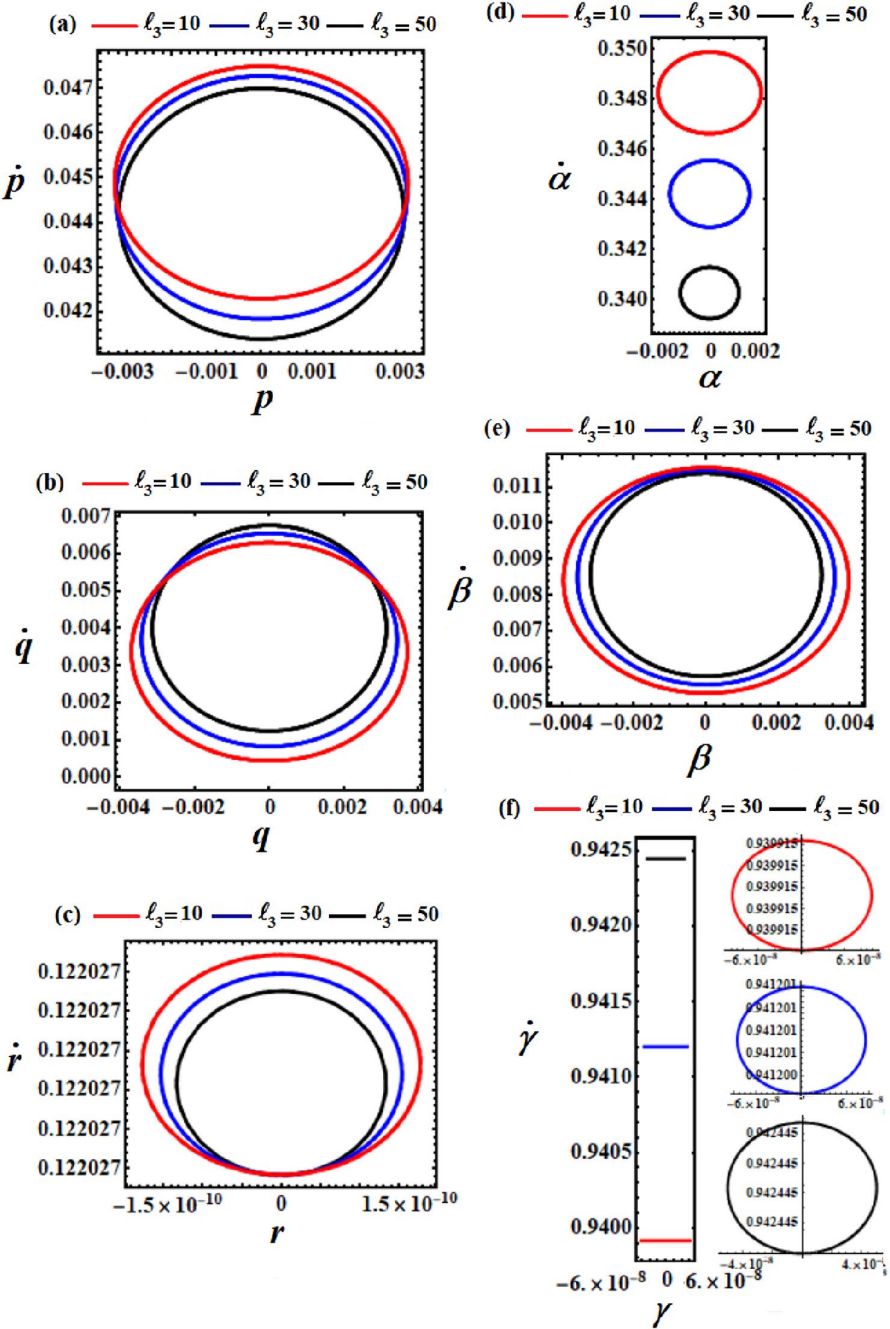
Displays the phase plane curves at $$\ell_{3} = (10,30,50)\,{\text{kg}}\,{\text{m}}^{2} \,{\text{s}}^{ - 1} ,$$$$\ell_{2} = 10\,{\text{kg}}\,{\text{m}}^{2} \,{\text{s}}^{ - 1} ,$$ and $$M = 100\,{\text{kg}}$$.

The variations of the obtained solutions $p,q,r,\alpha ,\beta ,$ and $\gamma$, corresponding to different values of $\ell_{3}$ (denoted as $10,30,$ and $50$), are depicted in parts of Fig. 4. All of these solutions are acted upon by the change of $\ell_{3}$ values, where periodic waves with different amplitudes are depicted in the parts of Fig. 3. These amplitudes decrease with the increase in $\ell_{3}$ values for the represented waves of each, except the solution $q$. The phase plane plots representing these waves are graphed in parts of Fig. 5, providing insight into the stable behavior of the results. In other words, the obtained solution exhibits stable behavior devoid of chaos.

**Figure 6:**
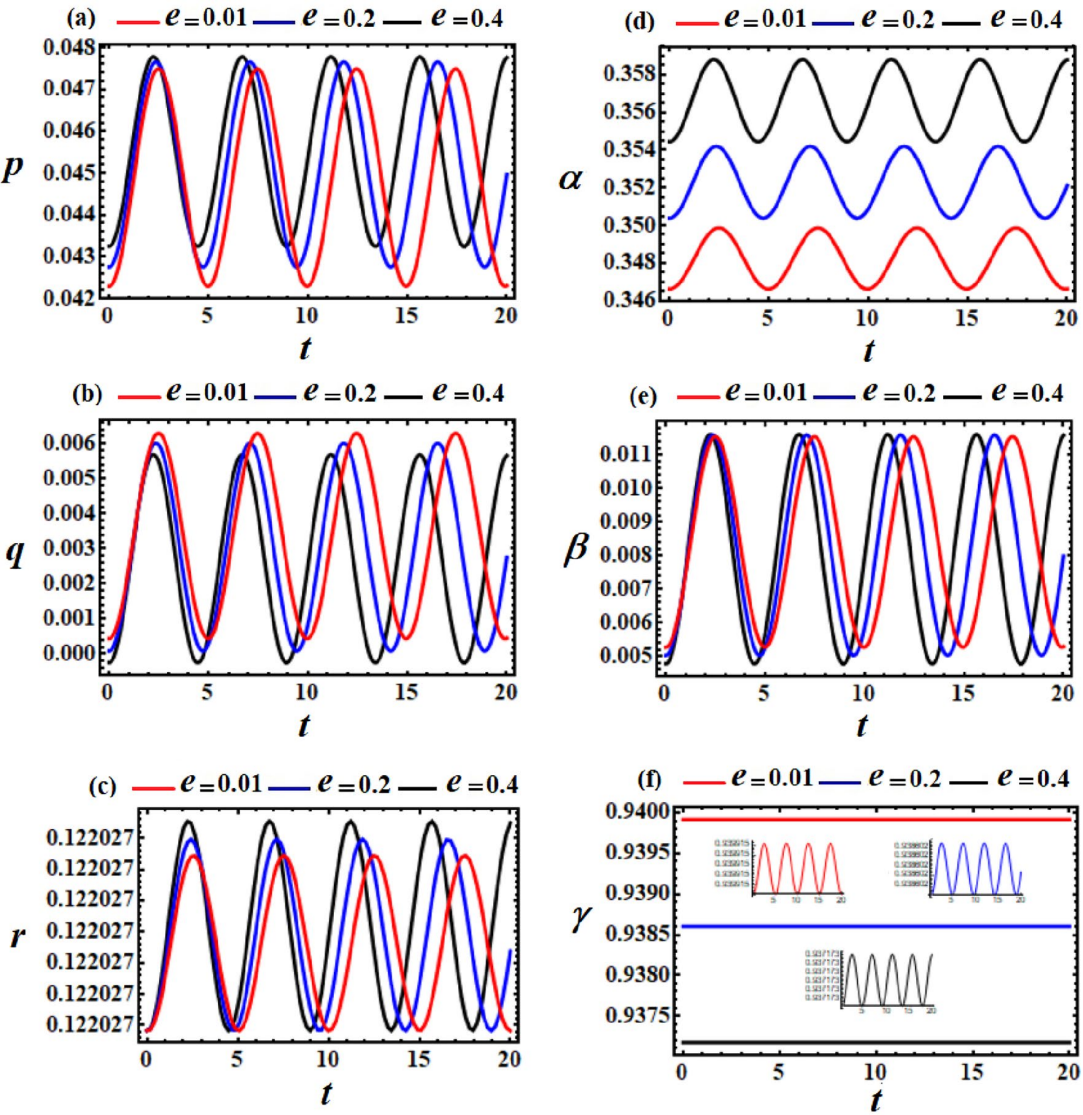
Phases represent the time histories of the obtained solutions at $\ell_{2} = \ell_{3} = 10\,{\text{kg}}\,{\text{m}}^{2} \,{\text{s}}^{ - 1}$ and $M = 100\,{\text{kg}}$.

**Figure 7:**
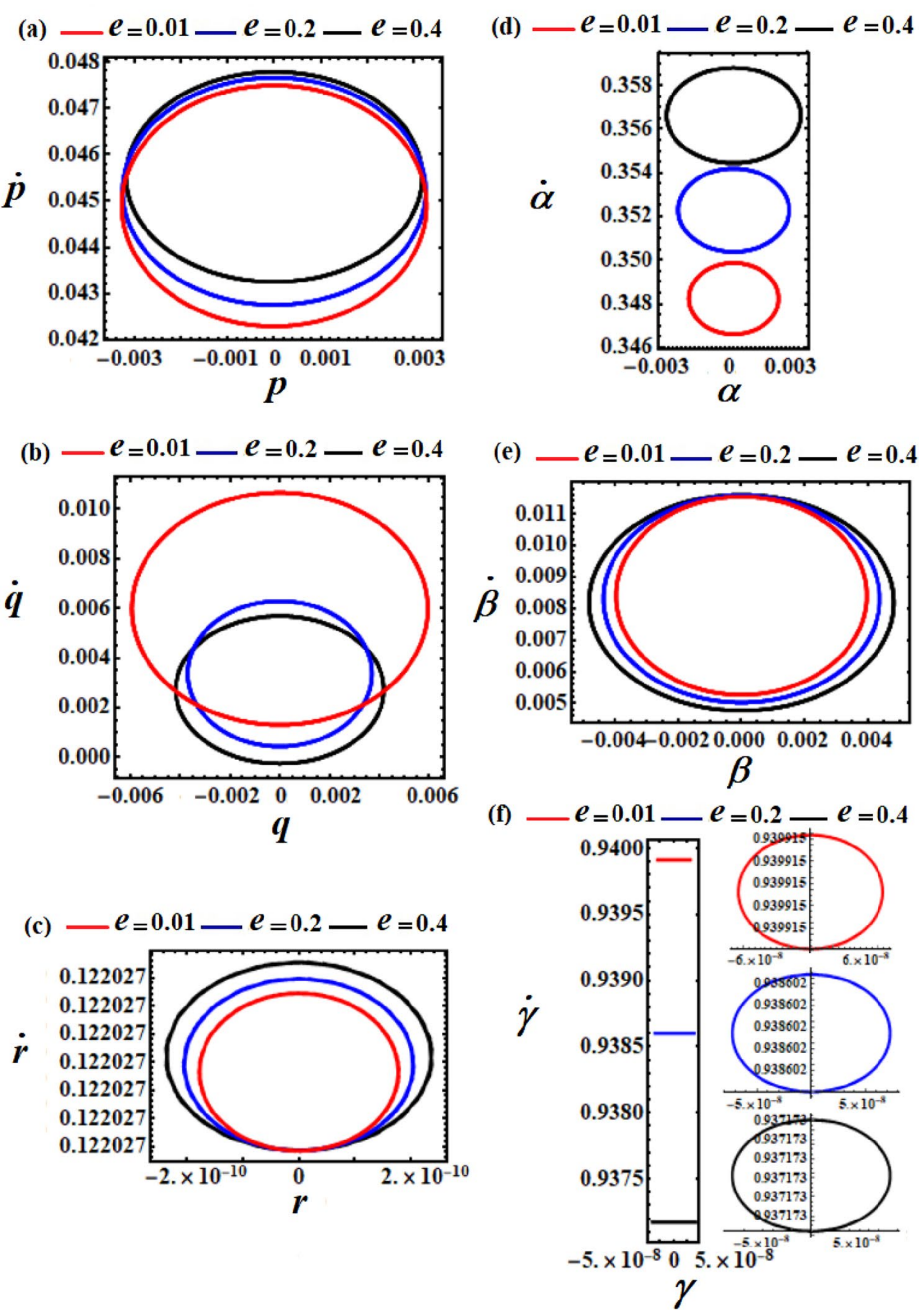
Sketches the phase portraits of the solutions $p,q,r,\alpha ,\beta ,$ and $\gamma$ at $\ell_{2} = \ell_{3} = 10\,{\text{kg}}\,{\text{m}}^{2} \,{\text{s}}^{ - 1}$ and $M = 100\,{\text{kg}}$.

The curves depicted in Figs. 6 and 7 were computed at $\ell_{2} = \ell_{3} = 10$ with the point charge $e( = 0.01,0.2,0.4)$. The amplitudes of the drawn periodic waves increase with the raise in $e$ values, as evident in portions (a), (c), (d), and (e) of Fig. 6. Conversely, for the solutions $q$ and $\gamma$, it’s noticeable that their wave amplitudes decrease as $e$ values increase, as sketched in Figs. 6b and f. This conclusion has been validated by plotting phase plane curves depicting the relationships between the achieved solutions and their corresponding first derivatives, see Fig. 7.

**Figure 8:**
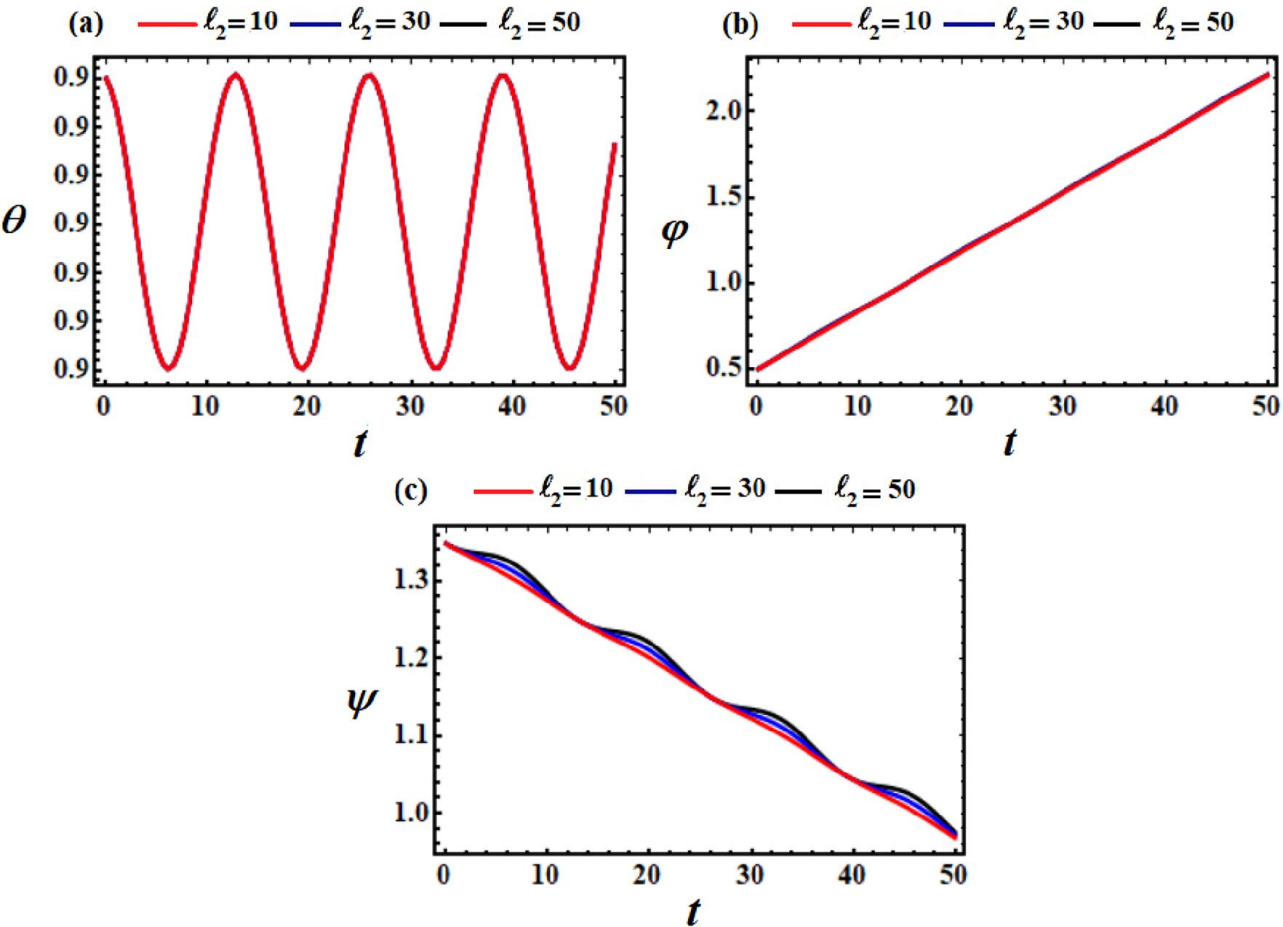
Demonstrates the temporal histories of the angles (**a**) $\theta ,$ (**b**) $\varphi ,$ and (**c**) $\psi ,$ at $\ell_{2} = (10,30,50)\,{\text{kg}}\,{\text{m}}^{2} \,{\text{s}}^{ - 1}$, and $\ell_{3} = 10\,{\text{kg}}\,{\text{m}}^{2} \,{\text{s}}^{ - 1}$ when $M = 100\,{\text{kg}}$.

**Figure 9:**
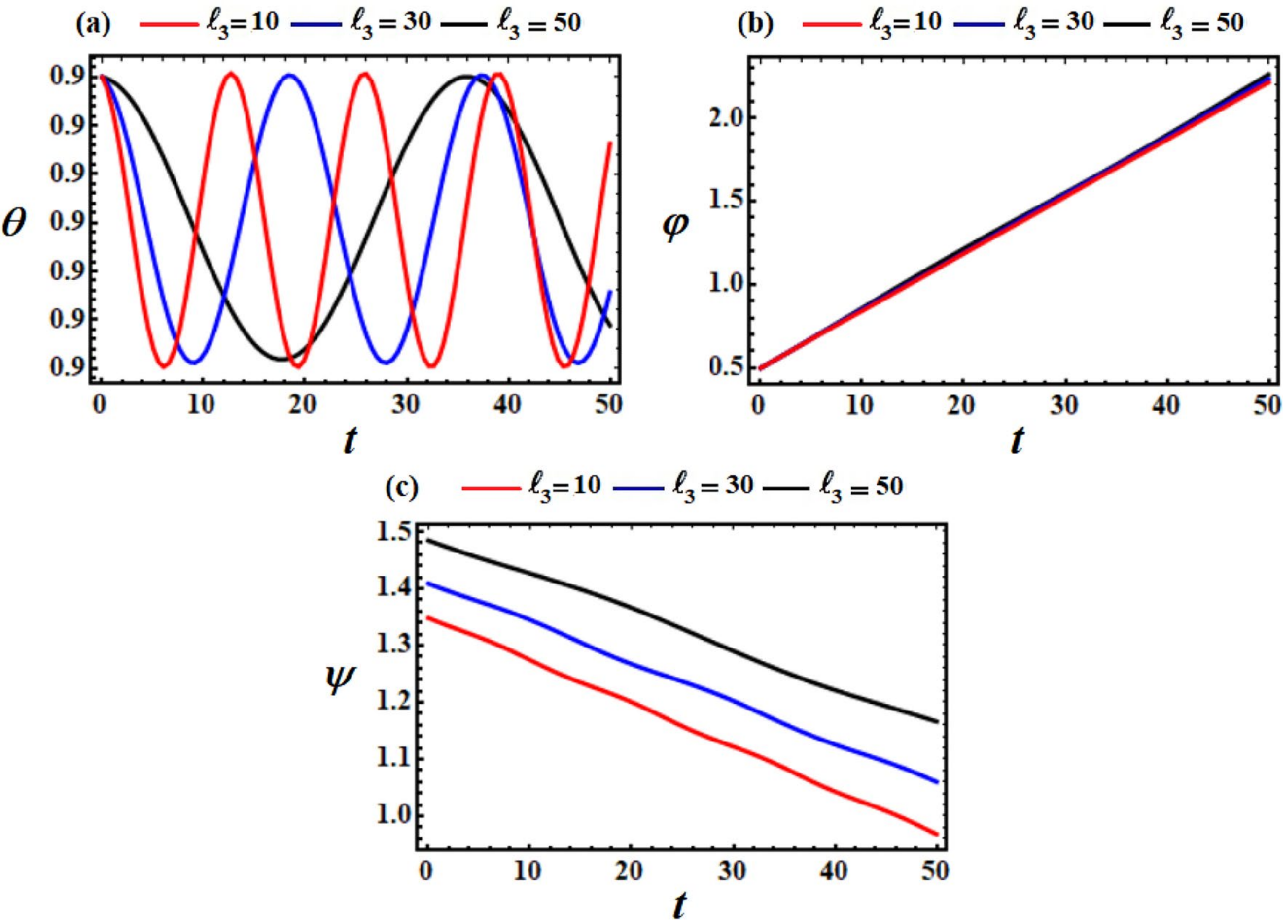
Demonstrates the temporal histories of the angles (**a**) $\theta ,$ (**b**) $\varphi ,$ and (**c**) $\psi ,$ at $\ell_{2} = 10\,{\text{kg}}\,{\text{m}}^{2} \,s^{ - 1}$, and $\ell_{3} = (10,30,50)\,{\text{kg}}\,{\text{m}}^{2} \,{\text{s}}^{ - 1}$ when $M = 100\,{\text{kg}}$.

**Figure 10:**
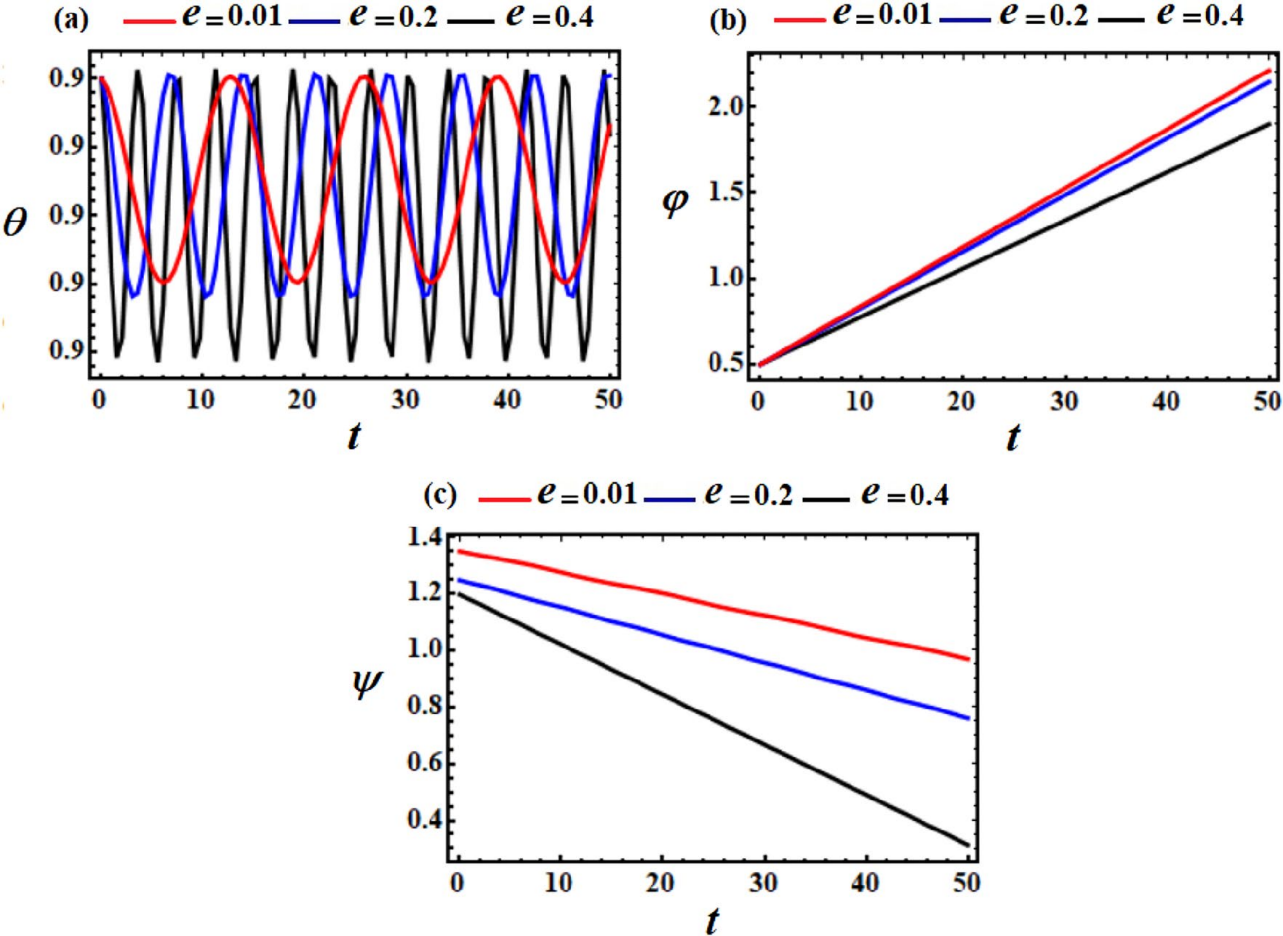
Explores the temporal histories of the angles (**a**) $\theta ,$ (**b**) $\varphi ,$ and (**c**) $\psi ,$ at $\ell_{2} = \ell_{3} = 10\,{\text{kg}}\,{\text{m}}^{2} \,{\text{s}}^{ - 1}$, and $e = (0.01,0.2,0.4)$ when $M = 100\,{\text{kg}}$.

It is noted that the curves in Figs. 8, 9, and 10 are considered a good advancement in figuring out the location and orientation of the body at any given time when $\ell_{2} ,\ell_{3} ,$ and $e$ have various values. To further elucidate these figures, let us proceed to examine their parts. Parts (a), (b), and (c) of these figures describe, respectively, the variation of the nutation $\theta$, the self-rotation $\varphi$, and the precession $\psi$ angles. The drawn curves for the angle of nutation $\theta$, have periodicity forms when the components of the GM $\underline{\ell }$ and the point charge $e$ vary, as seen in Figs. 8a, 9a and 10a.

However, the self-rotation angle $\varphi$ increases progressively as time goes on with the increase of $\ell_{2} ,\ell_{3} ,$ and $e$ values, as seen in Figs. 8b, 9b and 10b. This behavior can be attributed to the third term in the third equation of the system (39), wherein the magnitude of the first two terms is lower than that of the third term.

Conversely, the precession angle $\psi$ exhibits a decreasing fluctuation pattern over the analyzed time span, as depicted in Figs. 8c, 9c and 10c. On may state that, the reason of various initial points of these fluctuations is going back to the mathematical form of $\psi_{0}$ in Eq. (39).

## Conclusion

The dynamical rotatory movement of a charged symmetric RB, induced by a point charge positioned on its dynamic symmetry axis, has been examined, considering a slight displacement of the body’s center of mass from this axis. The analysis also incorporates the effects of two components of the GM about two of the body’s main axes of inertia, along with the impact of an electromagnetic field. The governing EOM associated with the equations of Euler–Poisson have been solved using PSPM, along with its modifications, particularly in a case involving irrational frequencies. The derived mathematical representations of Euler’s angles are utilized to determine the orientation or positioning of the body at different points in time. The newly acquired results have been plotted to better understand how the body moves over time, considering the defined parameter values. Phase plane graphs have been presented to illustrate the body’s stability throughout its motion. The novelty of examining the rotational dynamics of a charged RB in these specific conditions lies in the intricate interaction of gyrostatic effects, magnetic forces, and nonlinear dynamics. This research can extend the frontiers of theoretical mechanics and provide essential insights and tools for both academic progress and practical implementation. The obtained results can significantly improve the dynamic performance of various engineering applications, particularly those reliant on gyroscopic theory, like compasses, submarines, satellites, and aircraft automatic pilots.

### Supplementary Information

Supplementary Information.

## Data Availability

All data generated or analysed during this study are included in this published article.
