# Supplementary material for: The asymptotic solutions for the motion of a charged symmetric gyrostat in the irrational frequency case
Source: Sci Rep. 2024 Jul 19;14:16662. doi: 10.1038/s41598-024-66866-5 (PMC11271634; doi:10.1038/s41598-024-66866-5)
Supplement: Supplementary file 1 — Supplementary Information. [file 41598_2024_66866_MOESM1_ESM.doc]

**Appendix 1**

**Appendix 2**

$$\begin{matrix} \lambda_{1}^{-1}Q_{11}(T)=N_{10}+N_{11}M_{3}\sin\lambda_{2}T+N_{12}M_{3}\cos\lambda_{2}T+N_{13}\sin2\lambda_{1}T+N_{14}\cos2\lambda_{1}T \\ + N_{15}\sin2\lambda_{2}T+N_{16}\cos2\lambda_{2}T+N_{17}(M_{1}\sin\lambda_{1}T-M_{2}\cos\lambda_{1}T) \\ +N_{18}(M_{1}\sin\lambda_{1}T+M_{2}\cos\lambda_{1}T)+N_{19}(M_{1}\cos\lambda_{1}T-M_{2}\sin\lambda_{1}T) \\ +N_{110}(M_{1}\cos\lambda_{1}T+M_{2}\sin\lambda_{1}T)+N_{111}M_{3}[M_{1}\sin(\lambda_{1}-\lambda_{2})T \\ -M_{2}\cos(\lambda_{1}-\lambda_{2})T]+N_{112}M_{3}[M_{1}\sin(\lambda_{1}-\lambda_{2})T+M_{2}\cos(\lambda_{1}-\lambda_{2})T] \\ +N_{113}M_{3}[M_{1}\cos(\lambda_{1}-\lambda_{2})T-M_{2}\sin(\lambda_{1}-\lambda_{2})T]+N_{114}M_{3}[M_{1}\cos(\lambda_{1} \\ -\lambda_{2})T+M_{2}\sin(\lambda_{1}-\lambda_{2})T]+N_{115}M_{3}[M_{1}\sin(\lambda_{1}+\lambda_{2})T-M_{2}\cos(\lambda_{1} \\ +\lambda_{2})T]+N_{116}M_{3}[M_{1}\sin(\lambda_{1}+\lambda_{2})T+M_{2}\cos(\lambda_{1}+\lambda_{2})T]+N_{117}M_{3}[M_{1} \\ \times\cos(\lambda_{1}+\lambda_{2})T-M_{2}\sin(\lambda_{1}+\lambda_{2})T]+N_{118}M_{3}[M_{1}\cos(\lambda_{1}+\lambda_{2})T+M_{2} \\ \times\sin(\lambda_{1}+\lambda_{2})T]+N_{119}M_{3}^{2}[M_{1}\sin(\lambda_{1}-2\lambda_{2})T-M_{2}\cos(\lambda_{1}-2\lambda_{2})T] \\ +N_{120}M_{3}^{2}[M_{1}\sin(\lambda_{1}-2\lambda_{2})T+M_{2}\cos(\lambda_{1}-2\lambda_{2})T]+N_{121}M_{3}^{2}[M_{1} \\ \times\cos(\lambda_{1}-2\lambda_{2})T-M_{2}\sin(\lambda_{1}-2\lambda_{2})T]+N_{122}M_{3}^{2}[M_{1}\cos(\lambda_{1}-2\lambda_{2})T \\ +M_{2}\sin(\lambda_{1}-2\lambda_{2})T]+N_{123}\sin(2\lambda_{1}-\lambda_{2})T+N_{124}\cos(2\lambda_{1}-\lambda_{2})T, \end{matrix}$$

$$\begin{matrix} \lambda_{1}^{-1}Q_{21}(T)=N_{20}+N_{21}M_{3}\sin\lambda_{2}T+N_{22}M_{3}\cos\lambda_{2}T+N_{23}\sin2\lambda_{1}T+N_{24}\cos2\lambda_{1}T \\ + N_{25}\sin2\lambda_{2}T+N_{26}\cos2\lambda_{2}T+N_{27}(M_{1}\sin\lambda_{1}T-M_{2}\cos\lambda_{1}T) \\ +N_{28}(M_{1}\sin\lambda_{1}T+M_{2}\cos\lambda_{1}T)+N_{29}(M_{1}\cos\lambda_{1}T-M_{2}\sin\lambda_{1}T) \\ +N_{210}(M_{1}\cos\lambda_{1}T+M_{2}\sin\lambda_{1}T)+N_{211}M_{3}[M_{1}\sin(\lambda_{1}-\lambda_{2})T \\ -M_{2}\cos(\lambda_{1}-\lambda_{2})T]+N_{212}M_{3}[M_{1}\sin(\lambda_{1}-\lambda_{2})T+M_{2}\cos(\lambda_{1}-\lambda_{2})T] \\ +N_{213}M_{3}[M_{1}\cos(\lambda_{1}-\lambda_{2})T-M_{2}\sin(\lambda_{1}-\lambda_{2})T]+N_{214}M_{3}[M_{1}\cos(\lambda_{1} \\ -\lambda_{2})T+M_{2}\sin(\lambda_{1}-\lambda_{2})T]+N_{215}M_{3}[M_{1}\sin(\lambda_{1}+\lambda_{2})T-M_{2}\cos(\lambda_{1} \\ +\lambda_{2})T]+N_{216}M_{3}[M_{1}\sin(\lambda_{1}+\lambda_{2})T+M_{2}\cos(\lambda_{1}+\lambda_{2})T]+N_{217}M_{3}[M_{1} \\ \times\cos(\lambda_{1}+\lambda_{2})T-M_{2}\sin(\lambda_{1}+\lambda_{2})T]+N_{218}M_{3}[M_{1}\cos(\lambda_{1}+\lambda_{2})T+M_{2} \\ \times\sin(\lambda_{1}+\lambda_{2})T]+N_{219}M_{3}^{2}[M_{1}\sin(\lambda_{1}-2\lambda_{2})T-M_{2}\cos(\lambda_{1}-2\lambda_{2})T] \\ +N_{220}M_{3}^{2}[M_{1}\sin(\lambda_{1}-2\lambda_{2})T+M_{2}\cos(\lambda_{1}-2\lambda_{2})T]+N_{221}M_{3}^{2}[M_{1} \\ \times\cos(\lambda_{1}-2\lambda_{2})T-M_{2}\sin(\lambda_{1}-2\lambda_{2})T]+N_{222}M_{3}^{2}[M_{1}\cos(\lambda_{1}-2\lambda_{2})T \\ +M_{2}\sin(\lambda_{1}-2\lambda_{2})T]+N_{223}\sin(2\lambda_{1}-\lambda_{2})T+N_{224}\cos(2\lambda_{1}-\lambda_{2})T, \end{matrix}$$

$$\begin{matrix} \lambda_{2}^{-1}Q_{31}(T)=N_{30}+N_{31}M_{3}\sin\lambda_{2}T+N_{32}M_{3}\cos\lambda_{2}T+N_{33}\sin2\lambda_{1}T+N_{34}\cos2\lambda_{1}T \\ + N_{35}\sin2\lambda_{2}T+N_{36}\cos2\lambda_{2}T+N_{37}(M_{1}\sin\lambda_{1}T-M_{2}\cos\lambda_{1}T) \\ +N_{38}(M_{1}\sin\lambda_{1}T+M_{2}\cos\lambda_{1}T)+N_{39}(M_{1}\cos\lambda_{1}T-M_{2}\sin\lambda_{1}T) \\ +N_{310}(M_{1}\cos\lambda_{1}T+M_{2}\sin\lambda_{1}T)+N_{311}M_{3}[M_{1}\sin(\lambda_{1}-\lambda_{2})T \\ -M_{2}\cos(\lambda_{1}-\lambda_{2})T]+N_{312}M_{3}[M_{1}\sin(\lambda_{1}-\lambda_{2})T+M_{2}\cos(\lambda_{1}-\lambda_{2})T] \\ +N_{313}M_{3}[M_{1}\cos(\lambda_{1}-\lambda_{2})T-M_{2}\sin(\lambda_{1}-\lambda_{2})T]+N_{314}M_{3}[M_{1}\cos(\lambda_{1} \\ -\lambda_{2})T+M_{2}\sin(\lambda_{1}-\lambda_{2})T]+N_{315}M_{3}[M_{1}\sin(\lambda_{1}+\lambda_{2})T-M_{2}\cos(\lambda_{1} \\ +\lambda_{2})T]+N_{316}M_{3}[M_{1}\sin(\lambda_{1}+\lambda_{2})T+M_{2}\cos(\lambda_{1}+\lambda_{2})T]+N_{317}M_{3}[M_{1} \\ \times\cos(\lambda_{1}+\lambda_{2})T-M_{2}\sin(\lambda_{1}+\lambda_{2})T]+N_{318}M_{3}[M_{1}\cos(\lambda_{1}+\lambda_{2})T+M_{2} \\ \times\sin(\lambda_{1}+\lambda_{2})T]+N_{319}M_{3}^{2}[M_{1}\sin(\lambda_{1}-2\lambda_{2})T-M_{2}\cos(\lambda_{1}-2\lambda_{2})T] \\ +N_{320}M_{3}^{2}[M_{1}\sin(\lambda_{1}-2\lambda_{2})T+M_{2}\cos(\lambda_{1}-2\lambda_{2})T]+N_{321}M_{3}^{2}[M_{1} \\ \times\cos(\lambda_{1}-2\lambda_{2})T-M_{2}\sin(\lambda_{1}-2\lambda_{2})T]+N_{322}M_{3}^{2}[M_{1}\cos(\lambda_{1}-2\lambda_{2})T \\ +M_{2}\sin(\lambda_{1}-2\lambda_{2})T]+N_{323}\sin(2\lambda_{1}-\lambda_{2})T+N_{324}\cos(2\lambda_{1}-\lambda_{2})T, \end{matrix}$$

$$\begin{matrix} \lambda_{2}^{-1}Q_{41}(T)=N_{40}+N_{41}M_{3}\sin\lambda_{2}T+N_{42}M_{3}\cos\lambda_{2}T+N_{43}\sin2\lambda_{1}T+N_{44}\cos2\lambda_{1}T \\ + N_{45}\sin2\lambda_{2}T+N_{46}\cos2\lambda_{2}T+N_{47}(M_{1}\sin\lambda_{1}T-M_{2}\cos\lambda_{1}T) \\ +N_{48}(M_{1}\sin\lambda_{1}T+M_{2}\cos\lambda_{1}T)+N_{49}(M_{1}\cos\lambda_{1}T-M_{2}\sin\lambda_{1}T) \\ +N_{410}(M_{1}\cos\lambda_{1}T+M_{2}\sin\lambda_{1}T)+N_{411}M_{3}[M_{1}\sin(\lambda_{1}-\lambda_{2})T \\ -M_{2}\cos(\lambda_{1}-\lambda_{2})T]+N_{412}M_{3}[M_{1}\sin(\lambda_{1}-\lambda_{2})T+M_{2}\cos(\lambda_{1}-\lambda_{2})T] \\ +N_{413}M_{3}[M_{1}\cos(\lambda_{1}-\lambda_{2})T-M_{2}\sin(\lambda_{1}-\lambda_{2})T]+N_{414}M_{3}[M_{1}\cos(\lambda_{1} \\ -\lambda_{2})T+M_{2}\sin(\lambda_{1}-\lambda_{2})T]+N_{415}M_{3}[M_{1}\sin(\lambda_{1}+\lambda_{2})T-M_{2}\cos(\lambda_{1} \\ +\lambda_{2})T]+N_{416}M_{3}[M_{1}\sin(\lambda_{1}+\lambda_{2})T+M_{2}\cos(\lambda_{1}+\lambda_{2})T]+N_{417}M_{3}[M_{1} \\ \times\cos(\lambda_{1}+\lambda_{2})T-M_{2}\sin(\lambda_{1}+\lambda_{2})T]+N_{418}M_{3}[M_{1}\cos(\lambda_{1}+\lambda_{2})T+M_{2} \\ \times\sin(\lambda_{1}+\lambda_{2})T]+N_{419}M_{3}^{2}[M_{1}\sin(\lambda_{1}-2\lambda_{2})T-M_{2}\cos(\lambda_{1}-2\lambda_{2})T] \\ +N_{420}M_{3}^{2}[M_{1}\sin(\lambda_{1}-2\lambda_{2})T+M_{2}\cos(\lambda_{1}-2\lambda_{2})T]+N_{421}M_{3}^{2}[M_{1} \\ \times\cos(\lambda_{1}-2\lambda_{2})T-M_{2}\sin(\lambda_{1}-2\lambda_{2})T]+N_{422}M_{3}^{2}[M_{1}\cos(\lambda_{1}-2\lambda_{2})T \\ +M_{2}\sin(\lambda_{1}-2\lambda_{2})T]+N_{423}\sin(2\lambda_{1}-\lambda_{2})T+N_{424}\cos(2\lambda_{1}-\lambda_{2})T, \end{matrix}$$

**Appendix 3**
